# Knockdown of lncRNA ENST00000609755.1 Confers Protection Against Early oxLDL-Induced Coronary Heart Disease

**DOI:** 10.3389/fcvm.2021.650212

**Published:** 2021-05-21

**Authors:** Yi Sun, Shuna Huang, Chunyu Wan, Qishuang Ruan, Xiaoxu Xie, Donghong Wei, Guobo Li, Shaowei Lin, Huangyuan Li, Siying Wu

**Affiliations:** ^1^School of Public Health, Fujian Medical University, Fuzhou, China; ^2^Department of Clinical Research and Translation Center Office, The First Affiliated Hospital of Fujian Medical University, Fuzhou, China; ^3^Department of Orthopedics, Fujian Medical University Union Hospital, Fuzhou, China

**Keywords:** coronary heart disease, long non-coding RNA, environmental factors, oxidized low-density lipoprotein, human coronary artery endothelial cells

## Abstract

**Background:** This study investigated the association between long non-coding RNAs (lncRNAs) and coronary heart disease (CHD) and further elucidated the potential biological roles of lncRNAs in CHD pathogenesis.

**Methods:** A case-control study (590 patients and 590 controls) was conducted from February 2017 and March 2019 in Fuzhou, China. Environmental factors were investigated using questionnaires and physical examinations. Five representative lncRNAs were screened using lncRNA microarray (peripheral blood in 5 cases and 5 controls) and further verified by quantitative real-time polymerase chain reaction (peripheral blood leukocyte in 100 cases and 100 controls). Oxidized low-density lipoprotein (oxLDL) was used to induce a human coronary artery endothelial cell (HCAECs) injury model, and loss of function was used to elucidate the role of lncRNA ENST00000609755.1 (lnc-MICALL2-2) in oxLDL-induced HCAECs injury.

**Results:** A total of 320 lncRNAs were found dysregulated in CHD patients (fold change> 2, *p* < 0.05). The results of a discovery microarray, population verification and HCAEC experiments suggested the lnc-MICALL2-2 is upregulated in CHD subjects and in an oxLDL-induced HCAECs injury model. Conversely, lnc-MICALL2-2 inhibition *in vitro* attenuated the effects of oxLDL on HCAECs morphology, proliferation, and apoptosis.

**Conclusion:** Elevated expression of lnc-MICALL2-2 is an independent risk factor for CHD, and knockdown subsequently confers protection against early pathological processes of oxLDL-induced CHD.

## Introduction

Coronary heart disease (CHD) is an ischemic heart disease and leading cause of death globally (1). There remains a need for increased understanding of the pathogenesis of CHD and the identification of new targets for the early diagnosis and therapy of CHD patients. Currently, it is recognized that family history of CHD, tobacco and alcohol consumption, sedentary lifestyle, obesity, anxiety, and depression are all environmental risk factors for CHD (2–5). However, these external factors can only partially explain the etiology of CHD. In addition to environmental and genetic predisposing factors, mounting evidence has shown that epigenetic alteration may play pivotal roles in the progression of CHD (6, 7).

Long non-coding RNAs (lncRNAs) are non-coding RNAs that are >200 nucleotides in length and widely distributed in the nucleus and cytoplasm (8). Approximately 321 lncRNAs have been identified in the mouse myocardium, the profile of which has demonstrated significant changes during development compared to the quiescent adult stage (9). It has been experimentally demonstrated that many of these lncRNAs particularly play a role in the regulation of pluripotency and the activation of cardiac-specific genes (10). The proximity of lncRNAs to known vascular disease-susceptibility loci helped to identify lncRNAs such as ANRIL and MIAT, subsequently shown to be associated with CHD risk (11) and myocardial infarction (12), respectively. Further, microarray approaches have identified thousands of abnormally expressed lncRNAs during human or animal models of cardiomyopathy (13). Despite the deep implications of lncRNAs in cardiovascular disease, their precise roles and mechanisms are not yet understood, especially in CHD.

Recent studies have reported that lncRNAs play a critical role in regulation of diverse cellular processes, such as vascular endothelial cells (VECs) dysfunction, vascular smooth muscle cell (VSMCs) proliferation, and lipid metabolism (14–16). VECs remodeling is considered a pivotal first step in the pathogenesis of CHD (17). Evidence for the role of lncRNAs in VECs remodeling comes from the finding that VECs under hypoxic stress upregulate the lncRNA MALAT1, which suggest an angiogenic effect in ischemia (14). In another study, investigators report that the lncRNA TGFB2-OT1 regulates various miRNA targets which participate in autophagy and induce pro-inflammatory cytokines of VECs (18). Oxidized low density lipoprotein (oxLDL) can disrupt the growth and survival of human coronary artery endothelial cells (HCAECs) through a malondialdehyde dependent pathway involving methylation of the FGF2 promoter and repression of FGF2 transcription (19). This oxLDL-induced VECs dysfunction can occur at concentrations as low as 50–150 μg/ml (20–22). Using this model in human umbilical vein endothelial cells, investigators have profiled thousands of abnormally expressed lncRNA, including fold changes as high as ~87-fold upregulated and ~28-fold downregulated (23). In this study, we choose HCAECs as the tool cells, the main considerations are: (1) Coronary artery endothelial injury is the early stage of the development of CHD. (2) HCAECs are widely used as tool cells to explore oxidative damage including endothelial dysfunction caused by ox-LDL (24–26). (3) The primary HCAECs ox-LDL injury model used in this manuscript is closer to the pathological conditions of CHD.

In this study we hypothesize that lncRNAs can affect the pathogenesis of CHD by regulating the biological function of VECs. To explore this hypothesis and the underlying molecular roles of target lncRNAs, a discovery microarray was executed in a small cohort of CHD and non-CHD patient samples. The lncRNA ENST00000609755.1 (lnc-MICALL2-2) was identified and validated as a potential target in CHD patients. Subsequently, HCAECs were exposed to oxLDL *in vitro* as a preliminary injury model for the exploration of the biological roles of lnc-MICALL2-2 during the controlled, early pathogenesis of CHD. This investigation lays groundwork for the further examination of a novel lncRNA that is abnormally regulated and biologically relevant in CHD.

## Materials and Methods

### Study Population and Environmental Factors

A total of 590 CHD patients and 590 controls were enrolled between February 2017 and March 2019 from the First Affiliated Hospital of Fujian Medical University and Fujian Medical University Union Hospital, China. The included CHD patients met the following criteria: (a) must present cardiac catheterization-confirmed stenosis (≥50%) in >1 major coronary artery; or (b) have a documented history of myocardial infarction; or (c) have a documented history of coronary revascularization (either by PCI or CABG); or (d) be enrolled during the stable stage after acute myocardial infarction; or (e) present confirmed ST-segment elevation/depression in electrocardiogram readings. This study excluded subjects with congenital heart disease, valvular disease, cardiomyopathy, somatization disorder, renal or hepatic disease, or those with insufficient comprehension of the Mandarin language. Control subjects presented a medical history devoid of cardiovascular diseases and were matched with CHD cases according to age, gender, marital status, and education level. Control subjects were enrolled during routine physical health examinations at the hospital and had not undergone cardiac catherization. The current study was approved by the Biomedical Research Ethics Committee of Fujian Medical University. All enrolled patients provided written informed consent, and the study protocol conformed to the ethical guidelines of the Declaration of Helsinki (1975).

Following enrollment, face-to-face interviews were performed to collect environmental exposure history. Sessions were conducted by trained interviewers according to a structured questionnaire. Information collected included demographic characteristics, family history of CHD, lifestyle habits, and psychological and physiological indicators (Supplementary Methods).

### lncRNAs Microarray Analysis

RNAs obtained from peripheral blood samples of five CHD patients and five non-CHD subjects were extracted for microarray analysis which ensured that as many differential lncRNAs as possible could be found. The basic situation of the 10 object was shown in Supplementary Table 1 (age range 50–70 years). A fast total RNA extraction kit (Biotech, Beijing, China) was used to extract the total RNA according to the manufacturer's instructions. The extracted RNAs were digested, dephosphorylated, denatured, amplified, and labeled with Cy3-dCTP according to the manufacturer's specifications. GeneSpring software V13.0 (Agilent Technologies, Santa Clara, CA, USA) was used to analyze the lncRNA array results and to summarize and normalize the data. The thresholds for this array were fold change (FC) of ≥2 or ≤ -2 and *p* ≤ 0.05 was the significance standard of *t*-tests. Array data were log-2 transformed and median-centered by genes through the adjusted data function of CLUSTER 3.0 software. Subsequent hierarchical clustering with average linkage was performed.

### Quantitative Real-Time Polymerase Chain Reaction (qRT-PCR)

Total RNA from peripheral blood leukocytes in 100 CHD patients and 100 non-CHD controls (Table 1) was extracted by using TRIzol reagent (Invitrogen, California, USA). All total RNA was of high purity with OD260/280 in the range of 1.80–2.00. One percentage of formaldehyde denaturing gel electrophoresis was used to determine the integrity of the RNA. RNA extracts were quantified using a NanoDrop 2000 instrument (Thermo Scientific, USA). One microgram of RNA was reverse-transcribed using a PrimeScript RT Reagent Kit with gDNA Eraser (lncRNAs). Before performing' lncRNA detection, all were pre-amplified. The qRT-PCR was performed on a Light Cycler 480 real-time PCR system (Roche, Switzerland) with the SYBR® Premix Ex TaqTM II kit (Takara Bio Inc., Shiga, Japan) for 40 cycles. β-actin was used as internal control. LncRNA expression levels were calculated using the 2^−ΔΔCT^ method. Supplementary Table 2 lists the primers used for qRT-PCR, similar research methods have been applied in previous studies (27, 28).

**Table 1:** Baseline characteristics of study participants.

**Characteristics**	**Total population**			**Population for qRT-PCR**		
	**CHD (%) *n* = 590**	**Non-CHD (%)*n* = 590**	***p* value**	**CHD (%)*n* = 100**	**Non-CHD (%) *n* = 100**	***p-*value**
Gender			0.447			1.000
Male	320 (54.2)	333 (56.4)		52 (52.0)	52 (52.0)	
Female	270 (45.8)	257 (43.6)		48 (48.0)	48 (48.0)	
Age			0.866			1.000
<65	272 (46.1)	267 (45.3)		43 (43.0)	43 (43.0)	
≥65	318 (53.9)	323 (54.7)		57 (57.0)	57 (57.0)	
Marital status			0.679			1.000
Marriage	537 (91.0)	541 (91.7)		91 (91.0)	91 (91.0)	
Single and others	53 (9.0)	49 (8.3)		9 (9.0)	9 (9.0)	
Education level			0.684			0.116
Below primary school	260 (44.1)	373 (46.3)		46 (46.0)	58 (58.0)	
Middle school	261 (44.2)	255 (43.2)		35 (35.0)	32 (32.0)	
College or higher	69 (11.7)	62 (10.5)		19 (19.0)	10 (10.0)	

*CHD, coronary heart disease; qRT-PCR, quantitative real-time PCR*.

### HCAECs Culture and Identification

HCAECs was selected as the VECs model and cell culture medium was provided by Procell Life Science & Technology Co., Ltd (Wuhan, China). HCAECs were cultured in medium with 5% CO_2_ at 37°C. Cells were grown in monolayer and conventionally passaged when cell attachment rate reached 90%. An inverted microscope (Olympus Corporation, IX51, Japan) was used to observe cell morphology and growth daily.

Von Willebrand factor (vWF) is a protein factor released during synthesis of VECs. This protein not only participates in blood coagulation and thrombosis but also acts as a characteristic factor in identifying *in-vitro*-cultured VECs (29). HCAECs were stained with vWF by Jaffe method according to standard immunohistochemical staining technique. vWF was stained with Cy3 dye (red) and nuclei were counterstained with 4′,6-diamidino-2-phenylindole dye (blue).

### oxLDL Treatment of HCAECs

OxLDL plays an important role in HCAECs dysfunction and has been implicated in atherosclerotic heart disease and CHD, though its mechanisms are not fully known (19, 30). *In vitro*, oxLDL has been used on cultured HCAECs to model the formation of atherosclerosis, as it disrupts the growth and survival of HCAECs (19). OxLDL (Dalian Meilun Biotechnology, Dalian, China) was oxidized using Cu_2_SO_4_ (oxidant) in PBS. Oxidation is terminated by adding excess EDTA-Na_2_. Each lot was analyzed on agarose gel electrophoresis for migration vs. LDL. This lot of oxLDL migrated 2.0-fold further than the native LDL and its purity reached more than 98%. To determine the appropriate concentration and exposure length for oxLDL in the treatment of HCAECs, the density of HCAECs in normal logarithmic growth phase was adjusted to 2 × 10^5^ cells/ml and cultured in 96-well plates (3 plates, 12 holes per plate) during 3rd passage. HCAECs were incubated with different concentrations of oxLDL (0, 50, 100, and 150 μg /ml) for 24, 48, and 72 h, respectively.

### siRNA Transfection

Prior to transfection, HCAECs were seeded at a density of 5.0 × 10^5^ cells/ml, and 200 μL serum-free Opti-MEM was added into six-well plates. siRNA (Gemma Gene, Shanghai, China) was added at a final concentration of 50 nM. Three lnc-MICALL2-2-targeting siRNA (siRNA-1, siRNA-2 and siRNA-3, Supplementary Table 3), were transfected into HCAECs with LipofectamineTM 2000 (Invitrogen, Shanghai, China) according to the manufacturer's instructions. Knockdown was confirmed by assessing the expression of the lncRNA with qRT-PCR. After transfection with the interference fragment, the cells were treated with oxLDL (100 μg/ml) and incubated for 48 h at 37°C and 5% CO_2_ saturated humidity. The experimental groups were comprised of the following: (1) Normal HCAECs (untreated); (2) HCAECs treated with siRNA (siRNA group); (3) HCAECs treated with oxLDL (oxLDL group); and (4) HCAECs treated with oxLDL + siRNA (oxLDL + siRNA group) and three replicates were performed for each group.

### Transmission Electron Microscopy

HCAEC is processed as described above for 48 h in 6-well plates and processed for electron microscopy as follows: (1) The media were removed and the cells were fixed in 2.5% glutaraldehyde for 3–4 h at room temperature. (2) Rinse with 0.1 M phosphate buffer (PH7.4) 3 times for 15 min each time. (3) 1% osmium acid·0.1 M phosphate buffer (PH7.4) is fixed at room temperature (20°C) for 2 h. (4) Rinse with 0.1 M phosphate buffer (PH7.4) 3 times for 15 min each time. Next, Cell samples were dehydrated, infiltrated and embedded. The details are as follows: (1) Dehydration: 50–70–80–90–95–100–100% alcohol dehydration, 15 min each time. (2) Infiltration: acetone: 812 embedding agent = 1:1 mixed solution infiltrated overnight. Pure 812 embedding agent penetrated overnight. (3) Embedding: polymerize at 60°C for 48 h. Ultrathin sections (70–80 nm thick) were prepared and stained with uranium acetate and lead citrate. Cells were observed and imaged with a transmission electron microscope (HITACHI, HT7700-SS, Japan).

### Lactate Dehydrogenase (LDH) Release Assay

Supernatant of homogenized cells was used to measure LDH activity using a LDH kit (Built Bioengineering Institute, Nanjing, China) as suggested by the manufacturer. Absorbance of the solution was measured at 450 nm using a microplate reader (Thermo Scientific, MULTISKAN MK3, Beijing, China). And three replicates were performed for each group. Protein concentrations were calculated by standard controls provided in the kit.

### HCAECs Proliferation Assay

A total of 10 μl of Cell Counting Kit-8 (CCK8, Beyotime, Shanghai, China) solution was added to each well of cultured HCAECs, and the cells were incubated for 4 h at 37°C. Absorbance (optical density) was measured at 450 nm and the optical density was used to represent relative cell viability. Each group was represented by three parallel wells and runs were repeated three times for each group.

### HCAECs Apoptosis Assay

Apoptosis assay was performed using AnnexinV-APC-7-AAD Apoptosis Detection Kit (Kaikey Biological, Nanjing, China). Briefly, cells were collected after the indicated treatment, washed twice with cold phosphate-buffered saline, and treated with 500 μl binding buffer, 5 μl Annexin V-APC and 5 μl 7-AAD, followed by incubation at room temperature for 15 min in the dark. The number of cells on the machine was 10,000, and the sample was loaded at a low speed. According to the experimental cell conditions, the 7-AAD voltage was around 300, and the APC voltage was around 700. Cells were analyzed by flow cytometry to assess apoptosis (Beckman, CytoFLEX, Shanghai, China). Three runs were repeated for each group.

### Statistical Analyses

Distribution differences were examined by χ^2^-test for categorical variables or two-tailed Student's *t*-test for normally distributed data. LncRNA expression data were presented as mean ± standard deviation (SD). Univariate and multivariate unconditional logistic regression analyses were conducted to show the associations between environmental factors and risk of CHD. Statistically significant variables in univariate analysis were selected for further multivariate analysis. Statistically significant variables in multivariate analysis have been used as confounding factors in adjustments for the associations between lncRNAs and CHD. All tests were two-sided, and *p* < 0.05 was considered statistically significant. All statistical analyses were performed using SPSS 25.0 software.

## Result

### Study Population Demographics and Environmental Correlates

The CHD and non-CHD subjects consisted of 590 subjects each. No significant group differences were observed in age, gender, marital status, or education level (*p* > 0.05, Table 1), indicating the adequacy of frequency matching. Multivariate analysis revealed that patients in the CHD group were significantly more likely to have a family history of CHD [odds ratio (OR): 1.732; 95% confidence interval (CI): 1.174–2.557], a high-salt diet (OR: 1.543; 95% CI: 1.153–2.066), smoke tobacco (defined as at least 100 cigarettes during their lifetime) (OR: 2.487; 95% CI: 1.883–3.285), work in a light-intensity environment (classified by intensity, duration, and frequency of the participant's physical activity at work) vs. a moderate-intensity environment (OR: 1.573; 95% CI: 1.175–2.104), suffer from self-rated moderate to severe anxiety (OR: 2.802; 95% CI: 1.013–7.749) and have a BMI ≥ 24.0 (OR: 1.511; 95% CI: 1.166–1.985, Supplementary Table 4). Detailed criteria for environmental risk factors of CHD are found in Supplementary Methods.

### Identification and Validation of Peripheral Blood Lymphocytes Dysregulated lncRNAs

**Figure 1:**
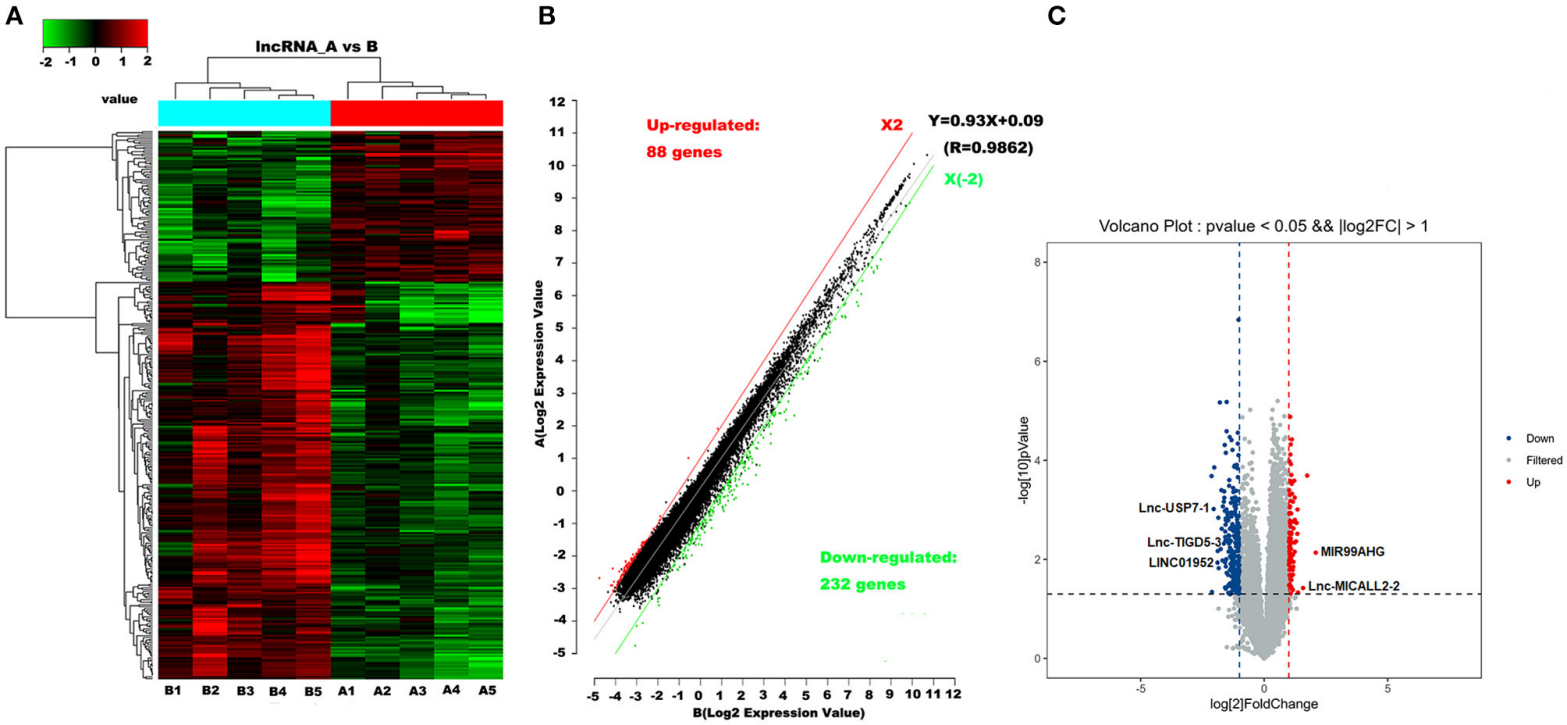
Differentially expressed lncRNAs in CHD vs. non-CHD subjects. **(A)** Heat map analysis from 10 samples in the CHD subjects (A1, A2, A3, A4, and A5) and non-CHD subjects (B1, B2, B3, B4, and B5) revealed different lncRNA expression profiles. The red color indicates relatively up-regulated lncRNAs, and the green color represents relatively down-regulated lncRNAs; the black portions indicate no significant difference. **(B,C)** Olcano plot and scatter plot analysis of differentially expressed lncRNAs in CHD and non-CHD subjects. Red or green points represent up-regulated or down-regulated lncRNAs (fold change >2.0, *p* < 0.05), respectively.

**Figure 2:**
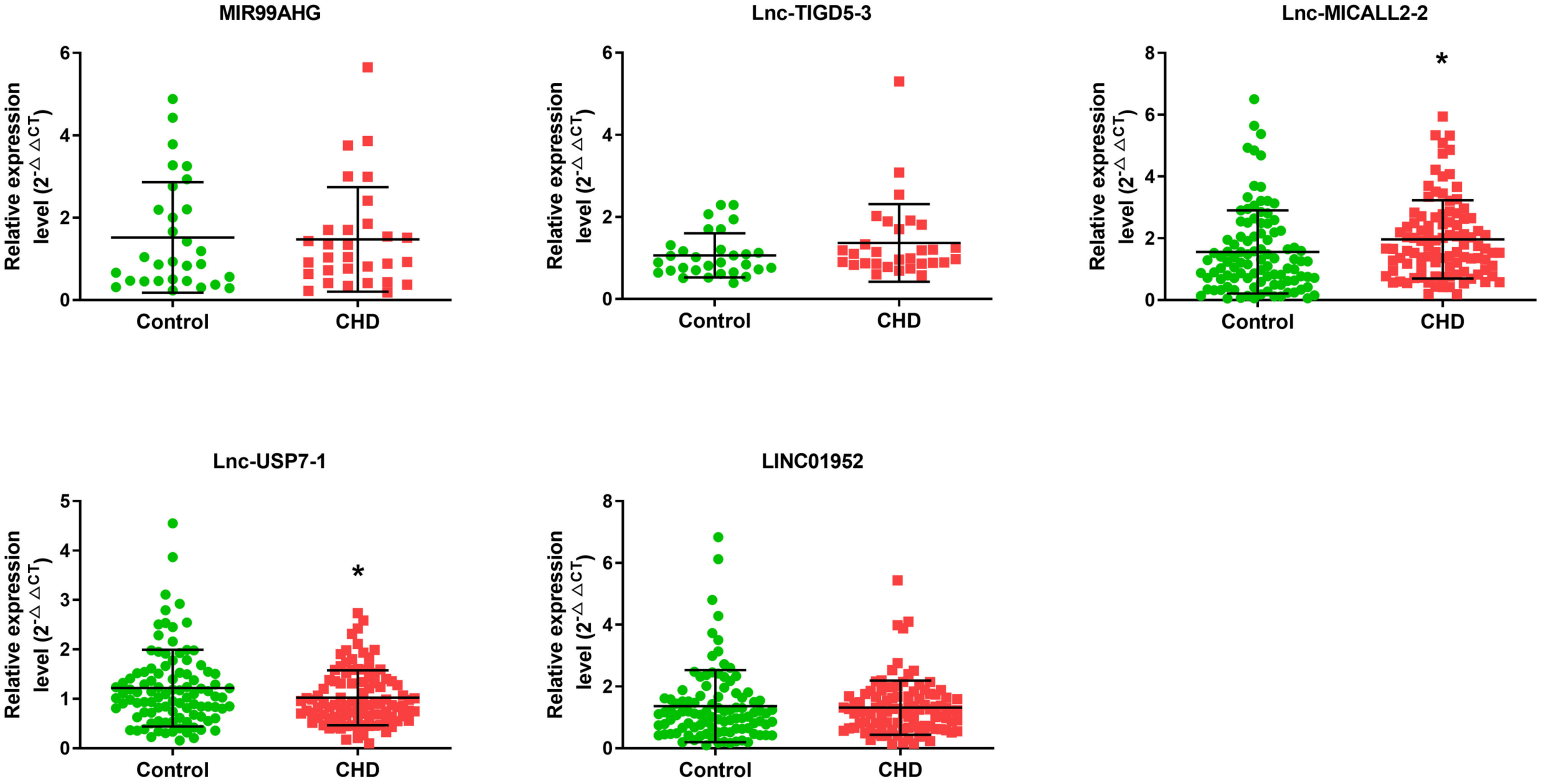
Comparison of the expression levels of lncRNAs between the CHD and non-CHD subjects. Five differentially expressed lncRNAs were validated by qRT-PCR. Data are expressed as means ± SD, (*n* = 30–100/group), *p < 0.05. Student *t*-test was used for all comparisons.

Microarray analyses revealed abnormal lncRNAs expression in the CHD cases provided for initial lncRNA identification (Figure 1A). The results of volcano plot and scatter plot indicated that a total of 88 and 232 lncRNAs were significantly up-regulated and significantly down-regulated in the CHD cases, respectively (FC >2, *p* < 0.05, Figures 1B,C). We chose 5 novel lncRNA candidates by conscientiously reviewing more stringent parameters (FC, *p*-value), variations between replicates and our previous study (28) (Supplementary Table 5 and Supplementary Figure 1). A larger population peripheral blood lymphocytes sample was subsequently used to validate the relative downregulation of lncRNA ENST00000565648.1 (lnc-USP7-1) in CHD subjects compared with non-CHD subjects, as well as the relative upregulation of lnc-MICALL2-2 (*p* < 0.05, Figure 2). These results agree with the findings of the microarray assay. After adjusting for environmental factors (age, gender, marital status, and education level, family history of CHD, high-salt diet, smoking, labor intensity, and BMI), lnc-USP7-1 and lnc-MICALL2-2 remained abnormally expressed in CHD patients compared to non-CHD controls (Table 2). On the other hand, no significant differences were observed between groups in the expression of lncRNAs ENST00000450016.1 (LINC01952), ENST00000602339.1 (MIR99AHG), and ENST00000529247.1 (lnc-TIGD5-3) (*p* > 0.05, Figure 2).

**Table 2:** Multivariable logistic regression analyses for the association between lncRNAs and CHD.

**Variable**	**Model-1^***a***^ OR (95% CI)**	**Model-2^***b***^ OR (95% CI)**
lnc-USP7-1	0.543 (0.332–0.890)	0.513 (0.295–0.891)
lnc-MICALL2-2	1.374 (1.080–1.747)	1.489 (1.077–2.057)

*CHD, coronary heart disease; OR, odds ratio; CI, confidence interval*.

a *Model-1: adjusted for age, gender, marital status, and education level*.

b *Model-2: adjusted for family history of CHD, high-salt diet, smoking, labor intensity, BMI, and the variables in Model-1*.

### Evaluation of the lncRNA Profiles of HCAECs Treated With oxLDL

**Figure 3:**
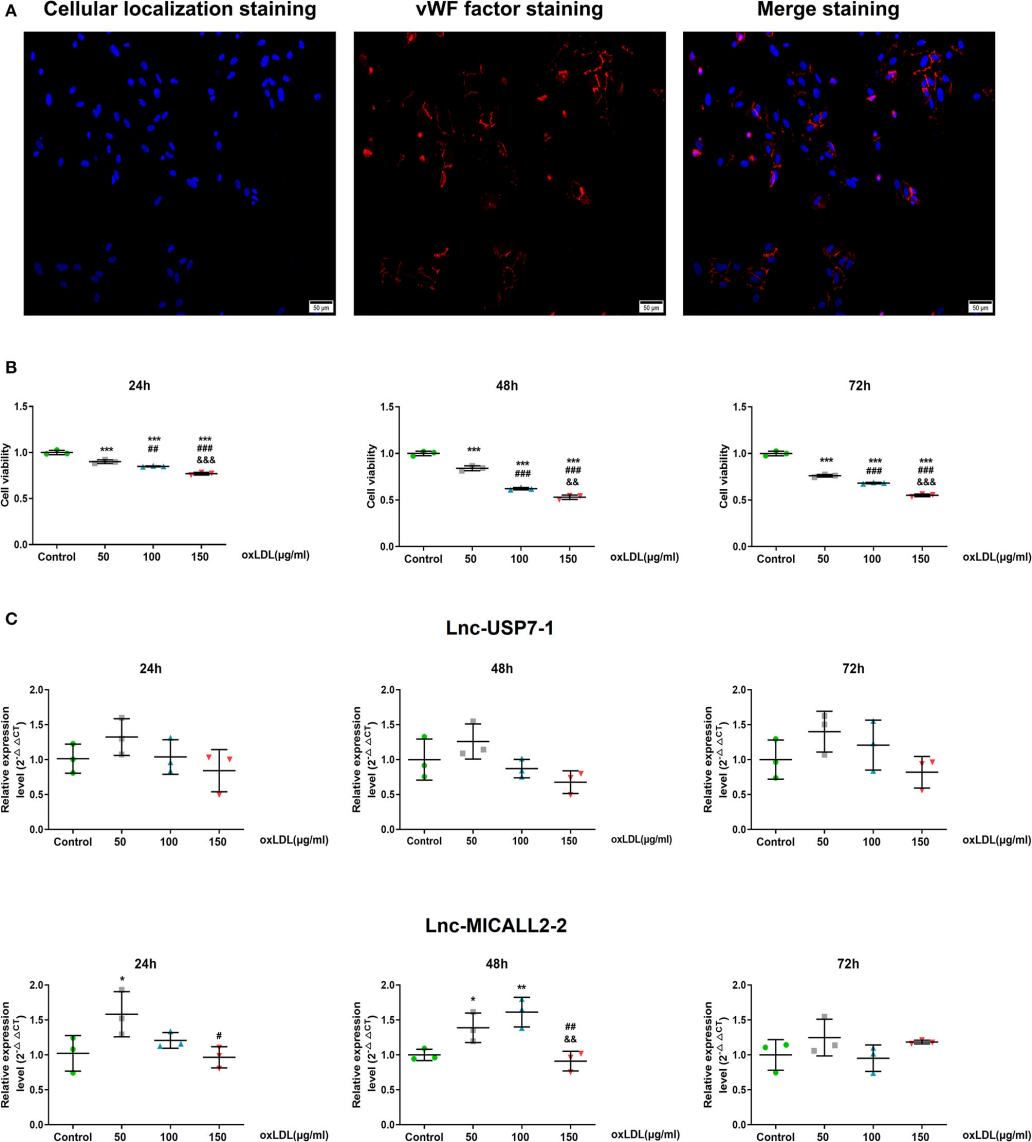
The expression levels of differentially expressed lncRNAs in HCAECs. **(A)** Purification identification of HCAECs by vWF. vWF factor (red) and cellular localization (blue) double staining ( ×200 times) of HCAECs. **(B)** Cell viabilities were compared after culturing at different concentrations of oxLDL for 24, 48, and 72 h (*n* = 3/group). **(C)** Comparison of the expression levels of differentially expressed lncRNAs after cell culture at different concentrations of oxLDL for 24, 48, and 72 h (*n* = 3/group). Values are expressed as the mean ± SD. *p < 0.05, **p < 0.01, ***p < 0.001 vs. normal HCAECs; ^#^*p* < 0.05, ^##^*p* < 0.01, ^###^*p* < 0.001 vs. 50 μg/ml oxLDL; ^&&^*p* < 0.01, ^&&&^*p* < 0.001 vs. 100 μg/ml oxLDL in panels b and c. Data are expressed as means ± SD. one-way ANOVA was used for all comparisons.

*In vitro* HCAECs experiments were further performed to better understand the role that these abnormally regulated lncRNAs may play in the etiology of CHD. First, almost all cells cultured were positive for the marker vWF, indicating that the culture of HCAECs yielded VECs with relatively high purity (Figure 3A). The application of oxLDL further revealed that HCAECs viability decreased in tandem with increasing concentrations of oxLDL. This phenomenon was true at exposures of 24, 48, and 72 h (Figure 3B). In contrast, no difference in the expression of the lnc-USP7-1 was observed between untreated HCAECs and oxLDL-treated HCAECs (*p* > 0.05). However, the expression of the lnc-MICALL2-2 was significantly upregulated in HCAECs treated with just 50 μg/ml oxLDL for 24 h compared to untreated controls. Interestingly, this effect was not observed at higher concentrations. The upregulation of the lncRNA was also observed at 50–100 μg/ml oxLDL at 48 h (Figure 3C, *p* < 0.05), though not in the 150 μg/ml oxLDL treated group. No differences in expression were apparent at any oxLDL dose after 72 h incubation. Based on these findings, the lnc-MICALL2-2 was selected for follow-up study, at a concentration of 100 μg/ml oxLDL for 48 h. This concentration and exposure was also of particular interest considering related studies (31).

### Knockdown of lnc-MICALL2-2 in oxLDL-Induced HCAECs Injury

**Figure 4:**
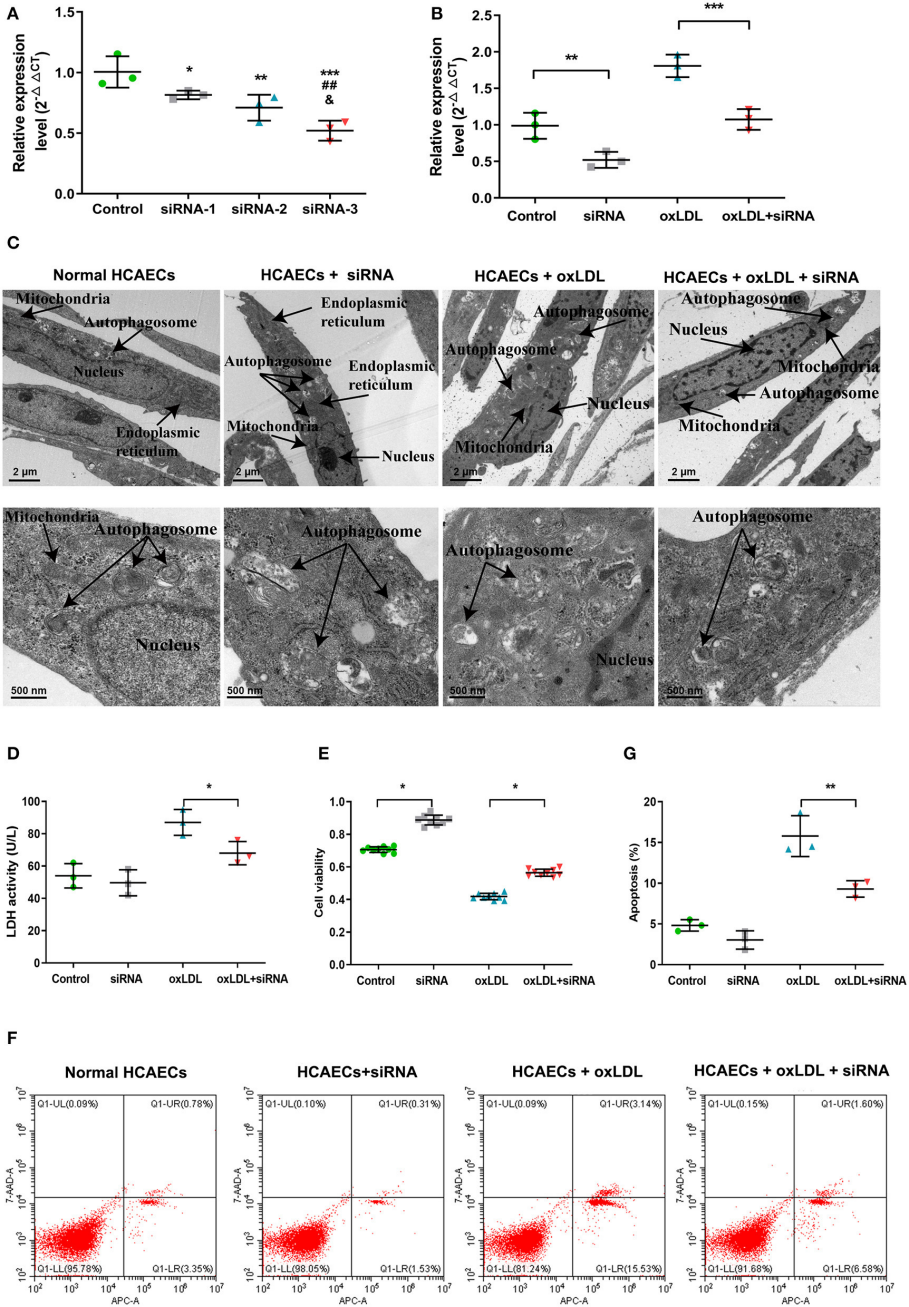
The function of knockdown of lnc-MICALL2-2 in oxLDL-induced HCAECs injury. **(A)** Lnc-MICALL2-2 interference fragment screening. *p < 0.05, **p < 0.01, ***p < 0.001 vs. normal HCAECs; ^##^*p* < 0.01 vs. HCAECs treated with siRNA-1; ^&^*p* < 0.05 vs. HCAECs treated with siRNA-2 (*n* = 3/group). **(B)** Relative expression of lnc-MICALL2-2 in normal HCAECs, HCAECs treated with siRNA, oxLDL, and oxLDL + siRNA. **(C)** HCAECs morphology of different treatment groups under electron microscopic observation (*n* = 3/group). **(D)** LDH activity in HCAECs culture medium of different treatment groups (*n* = 3/group). **(E)** HCAECs cell proliferation of different treatment groups activity by Cell Counting Kit-8 detection (*n* = 9/group). **(F,G)** HCAECs apoptosis of different treatment groups by flow cytometry (*n* = 3/group). Values are expressed as the mean ± SD, *p < 0.05, **p < 0.01, ***p < 0.001 in **(B,D,E,G)**. One-way ANOVA was used for all comparisons.

Due to the results of the lncRNA profile of oxLDL-induced HCAECs, which indicated the upregulation of lnc-MICALL2-2, the biological functions of the lncRNA was examined by knockdown with siRNA-3 (Figure 4A), which achieved an expression inhibition of ~50%. Consistent with prior results, cells treated with oxLDL observed a marked upregulation of the expression of the lnc-MICALL2-2. However, cells treated with siRNA-3 and oxLDL simultaneously saw normalization of the expression of the lncRNA compared with untreated cells (*p* < 0.05, Figure 4B).

Electron microscopy results revealed that untreated HCAECs nuclei were complete, with intact organelles presenting minimal autophagy. On the other hand, after treatment with 100 μg/mloxLDL, HCAECs showed nuclear lysis, organelle damage and severe autophagy (Figure 4C), demonstrated decreased LDH activity (Figure 4D), increased proliferation (Figure 4E), and decreased apoptosis (Figures 4F,G) in the oxLDL + siRNA group, illustrating that inhibition of lnc-MICALL2-2 attenuated the molecular markers of oxLDL injury in HCAECs.

## Discussion

CHD remains one of the leading causes of hospitalization and death worldwide, accounting for 64% of all cardiac deaths in 2009 (32). While the standard CHD medications (statins, anti-platelets, ACE inhibitors, beta blockers, etc.) greatly reduce the risk of complications arising from CHD, side-effects can greatly reduce quality of life (33). Evidence has shown that environmental factors, genomes, and epigenetics play crucial roles in it etiology (27). In this study, we confirmed that the elevated expression of lnc-MICALL2-2 is an independent risk factor for CHD after adjustment for environmental factors, and knockdown subsequently confers protection against early pathological processes of oxLDL-induced CHD.

The frequency of environmental characteristics observed in the CHD disease group recruited in this study were consistent with known CHD risk factors (family history, tobacco, moderate to severe anxiety, sedentary work environment, above average BMI) (34–38). This along with strict inclusion/exclusion criteria provided confidence that the group enrolled was representative of general CHD populations and provided evidence that controls were characteristically distinct from the disease group. Additionally, demographic profiling ensured that potentially confounding factors such as age, sex, or education level were equilibrated among the CHD and control groups. Though carried out in a relatively minute population of CHD patients, the discovery microarray reliably identified 320 differentially expressed lncRNAs in the disease group, providing a large pool of candidates for further study. After filtering the 320 lncRNAs, five were selected for validation due to fold change thresholds and predicted biological roles. Existing studies have shown that CHD is a chronic inflammatory disease. Inflammatory response plays an important role in the formation and development of atherosclerotic plaques and plaque rupture, and is one of the pathogenesis of CHD. The vascular inflammatory response involves complex interaction between leukocytes, endothelial cells (ECs), VSMCs, and extracellular matrix. Vascular injury is associated with increased expression of adhesion molecules by ECs and recruitment of leukocytes, growth factors, and cytokines (39). It has also been found that changes in the proportion of leukocytes in the blood in disease states can confound the aberrantly expressed signals observed in mixed-cell blood samples (40). Therefore, in this study, only comparative representative peripheral blood leukocytes were selected for lncRNA detection. Of these five, however, only two were consistent with the discovery array results. The lnc-USP7-1 was downregulated in CHD patients while lnc-MICALL2-2 was upregulated, even after adjusting for environmental factors. The implications of these findings were that these lncRNAs are strong candidates for CHD etiology and/or progression in CHD cohorts. In turn, these may be useful for diagnostic or therapeutic approaches in the future, though this work is novel and preliminary. In this study, results supported the hypothesis that novel lncRNAs are abnormally regulated in CHD patients. These findings are also consistent with the role of lncRNAs in VECs development and dysfunction (9, 14).

Oxidized lipid metabolites can activate platelet cascades and trigger thromboinflammatory factor release. Specifically, oxLDL binding of scavenger receptors on platelets can lead to production of reactive oxygen species, macrophage activation, and apoptosis. Further, platelet activation can induce chemokines which can promote inflammation and a pro-thrombotic microenvironment (41). While the dominant use of oxLDL *in vitro* has been used to establish models of atherosclerosis, considerable evidence has shown the successful induction of pre-atherosclerotic HCAECs dysfunction, death, and pathological neovascularization, even at low concentrations (42). Additional evidence for the involvement of oxLDL injury in CHD comes from the identification of serum oxLDL as a risk factor during early stage CHD in humans (43). Similarly, high serum levels of oxLDL receptor 1 have been associated with adverse events in patients with stable CHD (44) and in persons with high-calculated CHD risk prior to adverse events (45). Collectively, evidence substantiates the role of oxLDL challenge in the pathological transformation of VECs during the early etiology of CHD. In this study, it was further found that knockdown of the expression of lnc-MICALL2-2 *in vitro* attenuated the VECs damage caused by oxLDL, providing preliminary evidence for the role of a novel epigenetic target in the etiology of oxLDL-mediated CHD. Results from microarray assessments, population validation, and *in vitro* manipulation suggest that lnc-MICALL2-2 is not only a correlate of CHD development, but likely an influential factor in the etiology of the disease. In our previous bioinformatics analysis, lnc-MICALL2-2-related target genes were closely related to cell adherens junction, cadherin binding involved in cell adhesion, and focal adhesion signaling pathway is one of the top 10 highest signaling pathways (28), which is strongly associated with CHD (46).

VECs dysfunction is considered an early pathological process of CHD (17). Many findings have suggested that induction with different concentrations of ox-LDL (30–200 μg/ml) can cause inflammatory injury in HCAECs and produce an atherosclerotic phenotype, accompanied by increased expression levels of related inflammatory factors such as interleukin 6 (IL-6), IL-8 and tumor necrosis factor alpha (TNF-a) (26, 47, 48). Previous studies have shown that lncRNAs can be directly involved in the differentiation and proliferation of VECs or affect the VECs involved in immune regulation (49, 50). Notably, in this study inhibition of the lnc-MICALL2-2 significantly increased cell viability compared to normal cultured cells. Further, knockdown of lnc-MICALL2-2 in cells undergoing oxLDL challenge demonstrated improved viability, indicating that inhibition of the lncRNA not only increases HCAECs viability in general but attenuates the effect of oxLDL challenge on cell proliferation. This is in line with previous reports of the effects of oxLDL on HCAECs, which was in that study found to be mediated by the transcriptional repression of FGF2 (19). In addition, inhibition of lnc-MICALL2-2 attenuated oxLDL-induced HCAECs injury and apoptosis, particularly, nuclear lysis, organelle injury, and autophagy. There are many studies on the involvement of lncRNAs in the regulation of ox-LDL-induced apoptosis, and changes in their expression levels can affect the expression of related genes, which are involved in the regulation of the apoptotic process (26, 51, 52). It is foreseeable that the lnc-MICALL2-2 may similarly act on molecular targets that positively regulate oxLDL-induced cell death factors in HCAECs, though this remains to be thoroughly tested.

With mounting support for the use of many lncRNAs as molecular markers and therapeutic targets (53). Our study results showed that low expression of lnc-MICALL2-2 enhanced HCAECs proliferation and suppressed HCAECs apoptosis in an oxLDL-induced model of early CHD, which suggested that the knockdown of lnc-MICALL2-2 may become a future therapeutic approach for oxLDL-related CHD. LncRNAs typically exert their biological functions through interactions with regulatory proteins, miRNAs or other cellular factors (54–57). In our previous population-based research work, preliminary verification of lnc-MICALL2-2 potential ceRNA regulatory network in CHD. Although corresponding functional studies are still lacking, the results of this study further confirm that lnc-MICALL2-2 is indeed closely related to CHD, and deserves in-depth study (28). In addition, the LNCipedia database provides evidence that lnc-MICALL2-2 is not conserved in non-human species. This is consistent with the low conservation of lncRNA sequence, but the relatively high species specificity and tissue specificity (58).

Some limitations of this study include limited biological samples collected for microarray discovery. However, validation was performed in a relatively larger subset of participants, wherein two biologically relevant candidates were identified. Another limitation of this study was that the novel investigation of the lncRNA was performed in a cell model of HCAECs due to the preliminary nature of the investigation. In the future, it would be nice to see additional experiments beyond Figure 4 characterizing roles of lnc-MICALL2-2 on endothelial phenotypes or oxLDL-induced endothelial dysfunction, including the function of lncRNAs changes in other cell types such as macrophages or VSMCs. For example, are there changes in gene expression of inflammatory cytokines (e.g., TNF alpha) or adhesion molecules (e.g., ICAM-1, VCAM-1) when treating HCAECs with oxLDL or lnc-MICALL2-2 siRNA treatment? Finally, due to the exploratory nature of the study, an inhibitory technique that demonstrated partial repression of the lncRNA was selected for the investigation of the biological roles in an oxLDL model. In the future, targeted inhibition should be carried out in a dose-wise manner, allowing the investigation of the lncRNA at a full range (i.e. complete silencing, 25% expression, etc.) of expression levels.

In summary, elevated expression of lnc-MICALL2-2 is an independent risk factor for CHD, and knockdown subsequently confers protection against early pathological processes of oxLDL-induced CHD. This work presents novel evidence that lnc-MICALL2-2 may play a role in the etiology CHD. These findings lay a preliminary groundwork for a more detailed understanding of the molecular mechanisms of lnc-MICALL2-2 in the future.

## Data Availability Statement

The datasets presented in this study can be found in online repositories. The names of the repository/repositories and accession number(s) can be found at: NCBI [accession: GSE169256].

## Ethics Statement

The studies involving human participants were reviewed and approved by Biomedical Research Ethics Committee of Fujian Medical University. The patients/participants provided their written informed consent to participate in this study.

## Author Contributions

SW, HL, and SL contributed to the study design and helped to revised the manuscript. YS, SH, CW, and QR involved in writing and review of the manuscript. SH and SL conducted statistical analysis. YS, CW, XX, DW, and GL contributed to data collection and laboratory test. All authors contributed to critical revision of the final manuscript and approved the final version of the manuscript. The financial support and study supervision were provided by SW, HL, and SL.

## Conflict of Interest

The authors declare that the research was conducted in the absence of any commercial or financial relationships that could be construed as a potential conflict of interest.

## Abbreviations

CHD: Coronary heart disease
LncRNAs: Long non-coding RNAs
VECs: Vascular endothelial cells
VSMCs: Vascular smooth muscle cells
Ox-LDL: Oxidized low density lipoprotein
HCAECs: Human coronary artery endothelial cells
HCEAC: human coronary artery endothelial cell
FC: Fold change
qRT-PCR: Quantitative real-time polymerase chain reaction
vWF: Von Willebrand factor
SD: Standard deviation
OR: Odds ratio
CI: Confidence interval
BMI: Body mass index
IL-6: Interleukin 6
TNF-α: Tumor necrosis factor alpha
ECs: Endothelial cells.
