# Supplementary material for: Knockdown of lncRNA ENST00000609755.1 Confers Protection Against Early oxLDL-Induced Coronary Heart Disease
Source: Front Cardiovasc Med. 2021 May 21;8:650212. doi: 10.3389/fcvm.2021.650212 (PMC8175657; doi:10.3389/fcvm.2021.650212)
Supplement: Supplementary file 1 [file Data_Sheet_1.ZIP › raw data/Fig.4/Flow Cytometry(f).docx]

实验内容：细胞流式凋亡检测

**1.正常对照组（3组重复）（设定好阴性对照后，将处理的细胞上机检测通常是10000个细胞）**


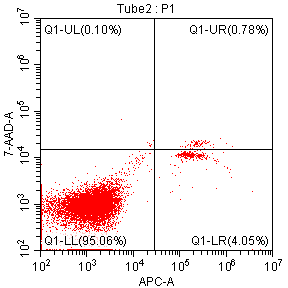


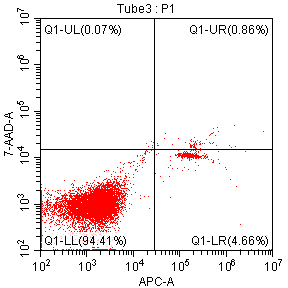


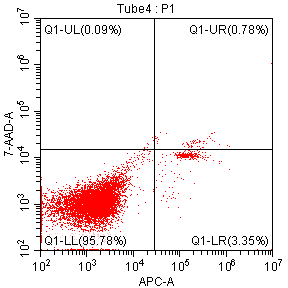


由上述分析方式可得正常组凋亡率为：（3个生物重复）

|  | 1 | 2 | 3 |
| --- | --- | --- | --- |
| 正常组 | 0.78%+4.05% | 0.86%+4.66% | 0.78%+3.35% |

**2. ENST00000609755.1-homo-2194组（3组重复）**


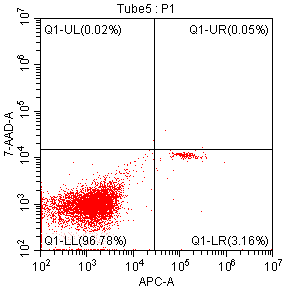


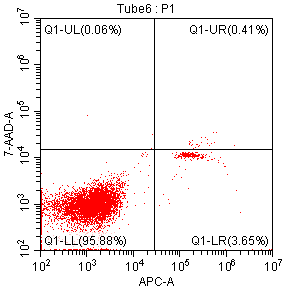


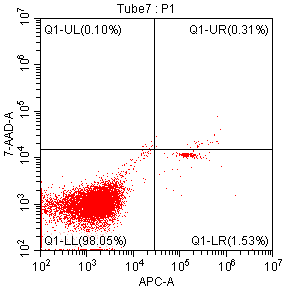


由上述分析方式可得干扰组凋亡率为：（3个生物重复）

|  | 1 | 2 | 3 |
| --- | --- | --- | --- |
| 干扰组 | 0.05%+3.16% | 0.41%+3.65% | 0.31%+1.53% |

**3.oxLDL组（3组重复）**

**
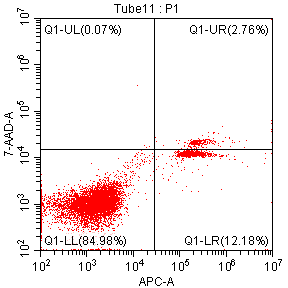
**

**
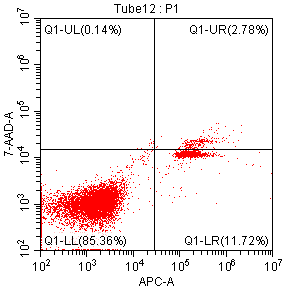
**

**
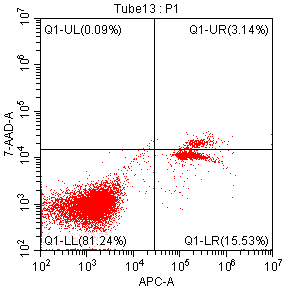
**

由上述分析方式可得**oxLDL**组凋亡率为：（3个生物重复）

|  | 1 | 2 | 3 |
| --- | --- | --- | --- |
| **oxLDL**组 | 2.76%+12.18% | 2.78%+11.72% | 3.14%+15.53% |

**4. oxLDL组+ ENST00000609755.1-homo-2194组（3组重复）**


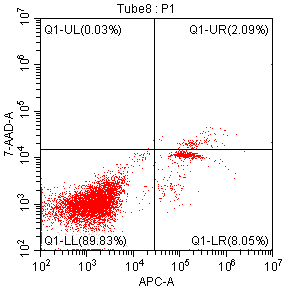


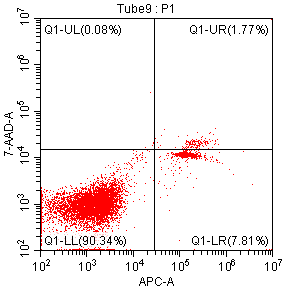


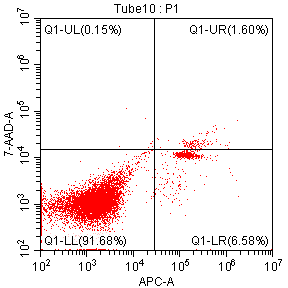


由上述分析方式可得**oxLDL+干扰**组凋亡率为：（3个生物重复）

|  | 1 | 2 | 3 |
| --- | --- | --- | --- |
| **oxLDL+干扰**组 | 2.09%+8.05% | 1.77%+7.81% | 1.60%+6.58% |

综上：

|  | 1 | | 2 | | 3 | |
| --- | --- | --- | --- | --- | --- | --- |
| 正常组 | 0.78%+4.05% | 4.83% | 0.86%+4.66% | 5.52% | 0.78%+3.35% | 4.13% |
| 干扰组 | 0.05%+3.16% | 3.21% | 0.41%+3.65% | 4.06% | 0.31%+1.53% | 1.84% |
| **oxLDL**组 | 2.76%+12.18% | 14.18% | 2.78%+11.72% | 14.5% | 3.14%+15.53% | 18.67% |
| **oxLDL+干扰**组 | 2.09%+8.05% | 10.14% | 1.77%+7.81% | 9.57% | 1.60%+6.58% | 8.18% |
