# Supplementary figures and images for: Knockdown of lncRNA ENST00000609755.1 Confers Protection Against Early oxLDL-Induced Coronary Heart Disease
Source: Front Cardiovasc Med. 2021 May 21;8:650212. doi: 10.3389/fcvm.2021.650212 (PMC8175657; doi:10.3389/fcvm.2021.650212)

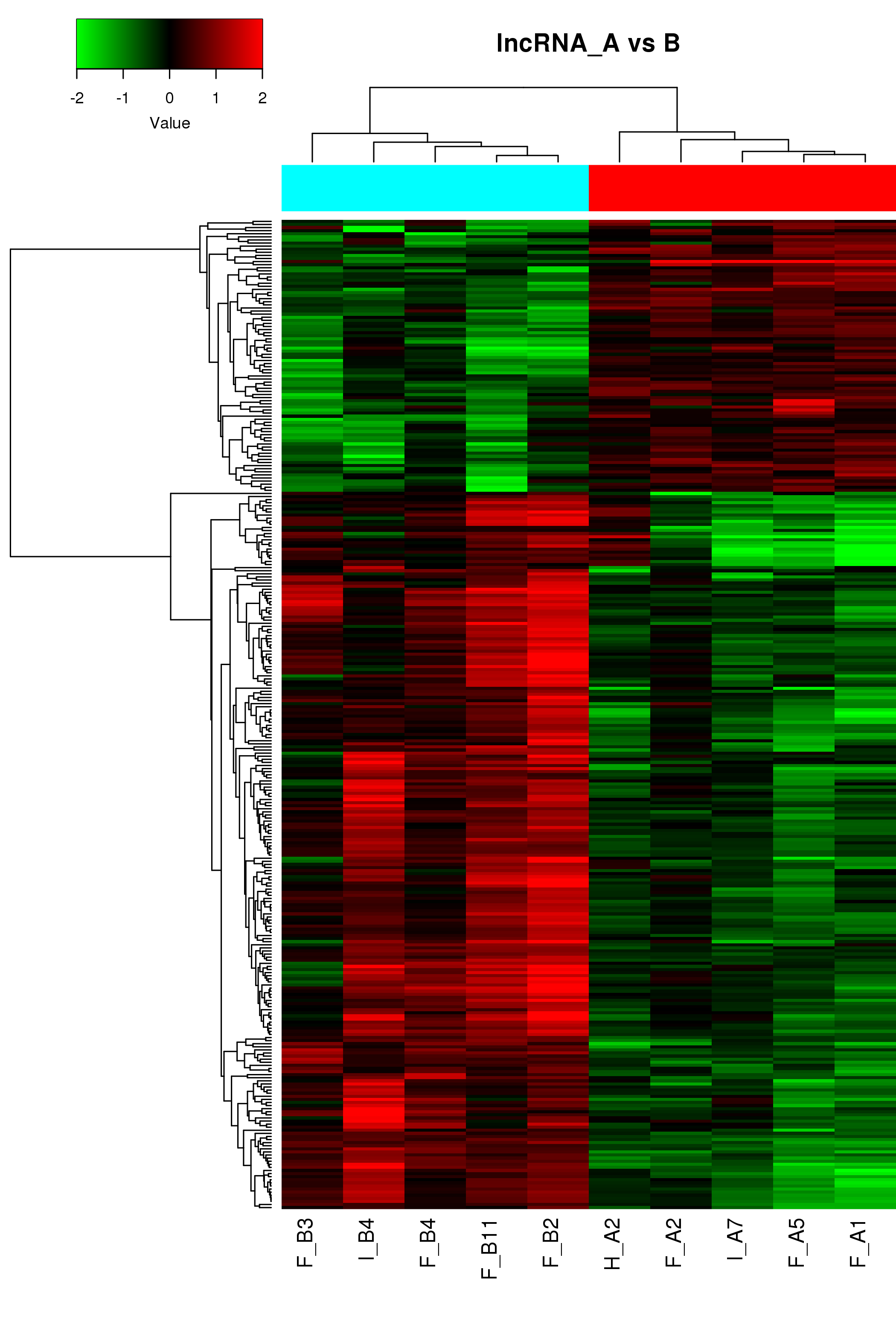

Supplement: Supplementary file 1 [file Data_Sheet_1.ZIP › raw data/Fig.1/cluster_lncRNA_A vs B.png]

# Scatter Plot

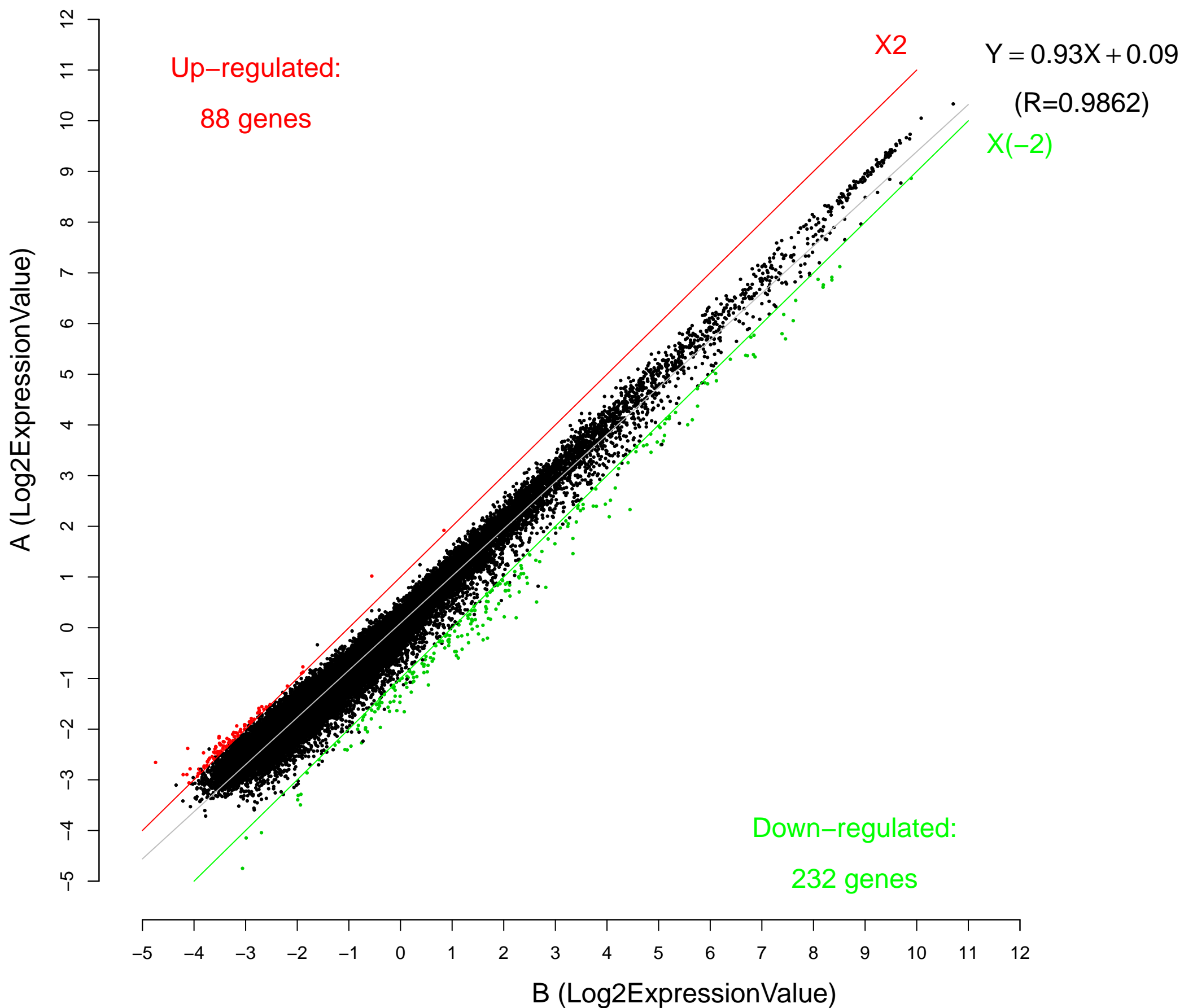

Supplement: Supplementary file 1 [file Data_Sheet_1.ZIP › raw data/Fig.1/scatter_function_lncRNA_A vs B.pdf]

# Volcano Plot

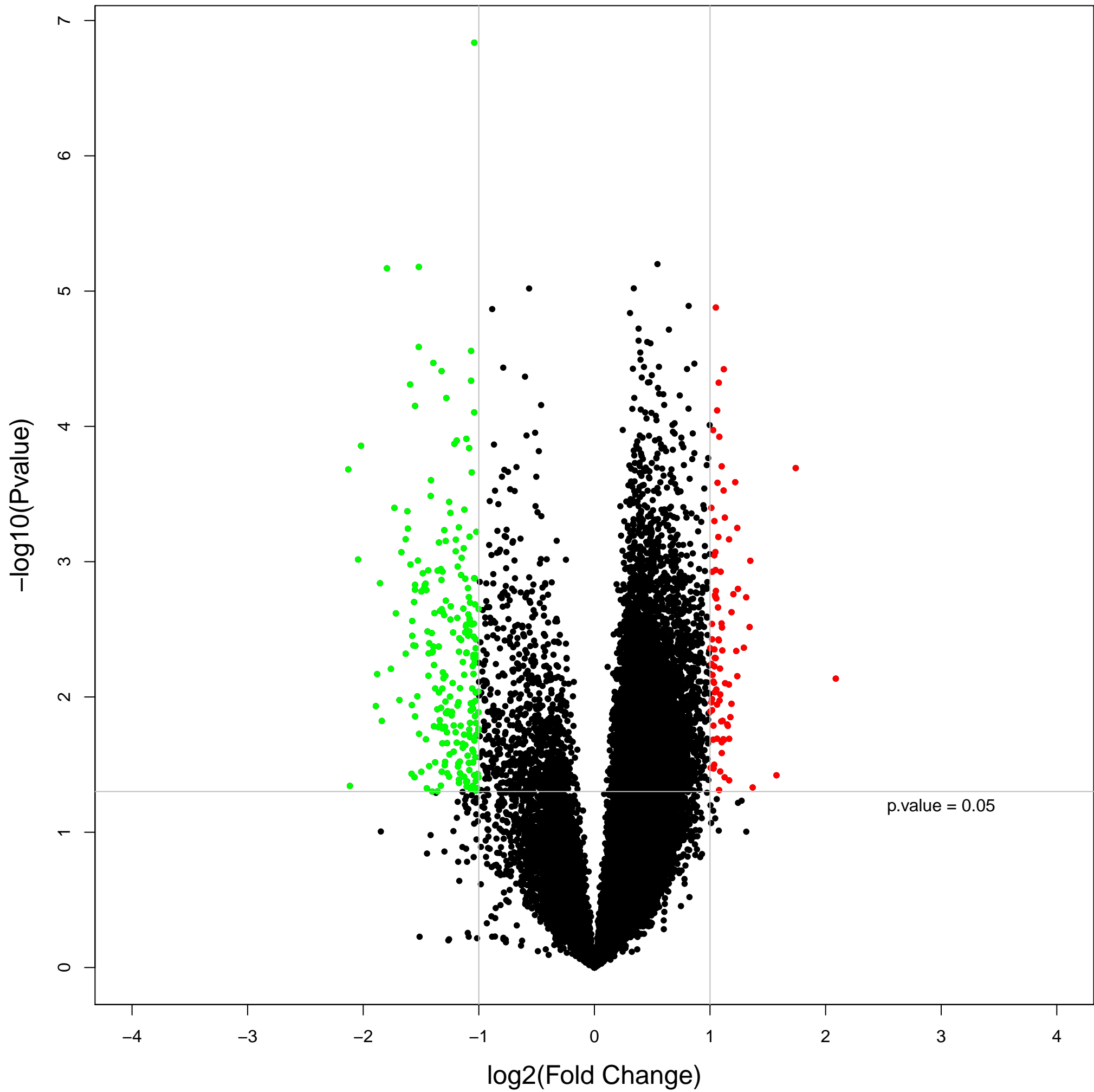

Supplement: Supplementary file 1 [file Data_Sheet_1.ZIP › raw data/Fig.1/volcano_lncRNA_A vs B.pdf]

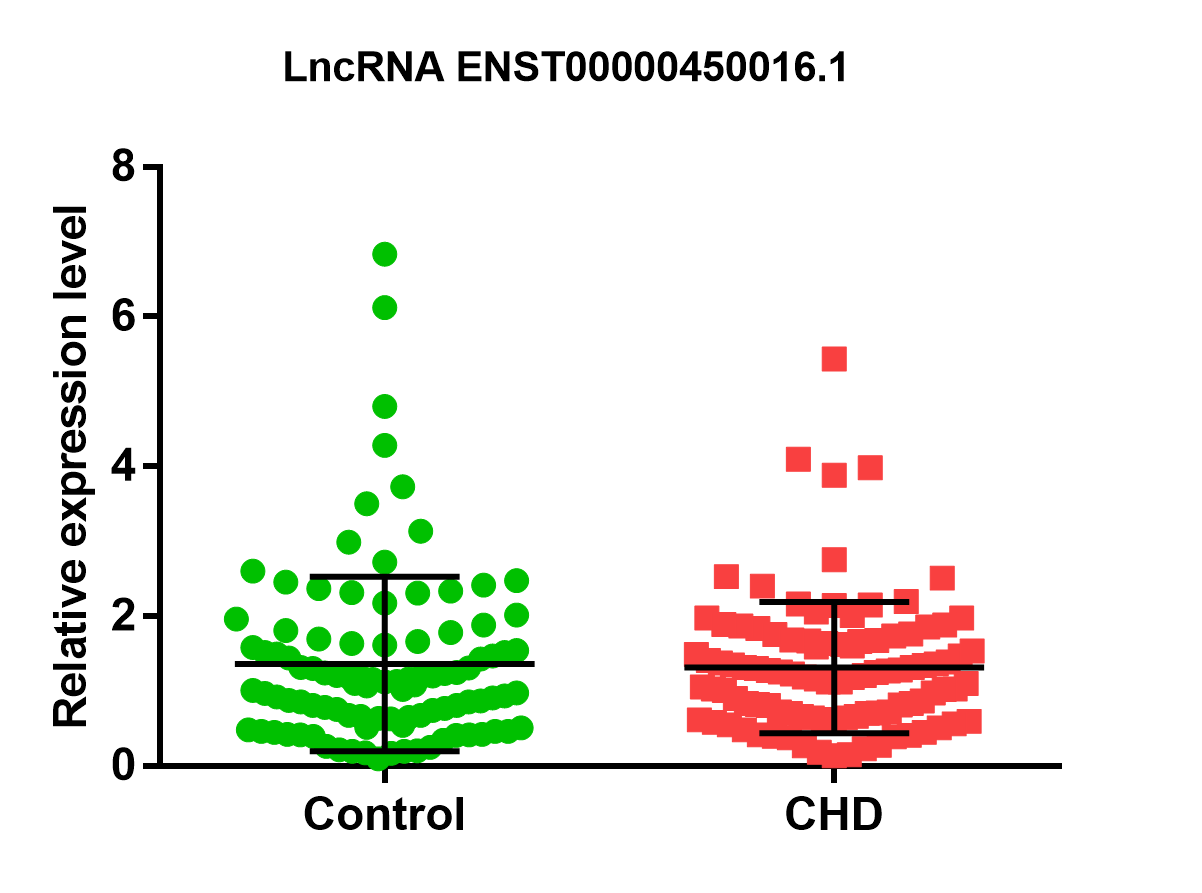

Supplement: Supplementary file 1 [file Data_Sheet_1.ZIP › raw data/Fig.2/161.1.tif]

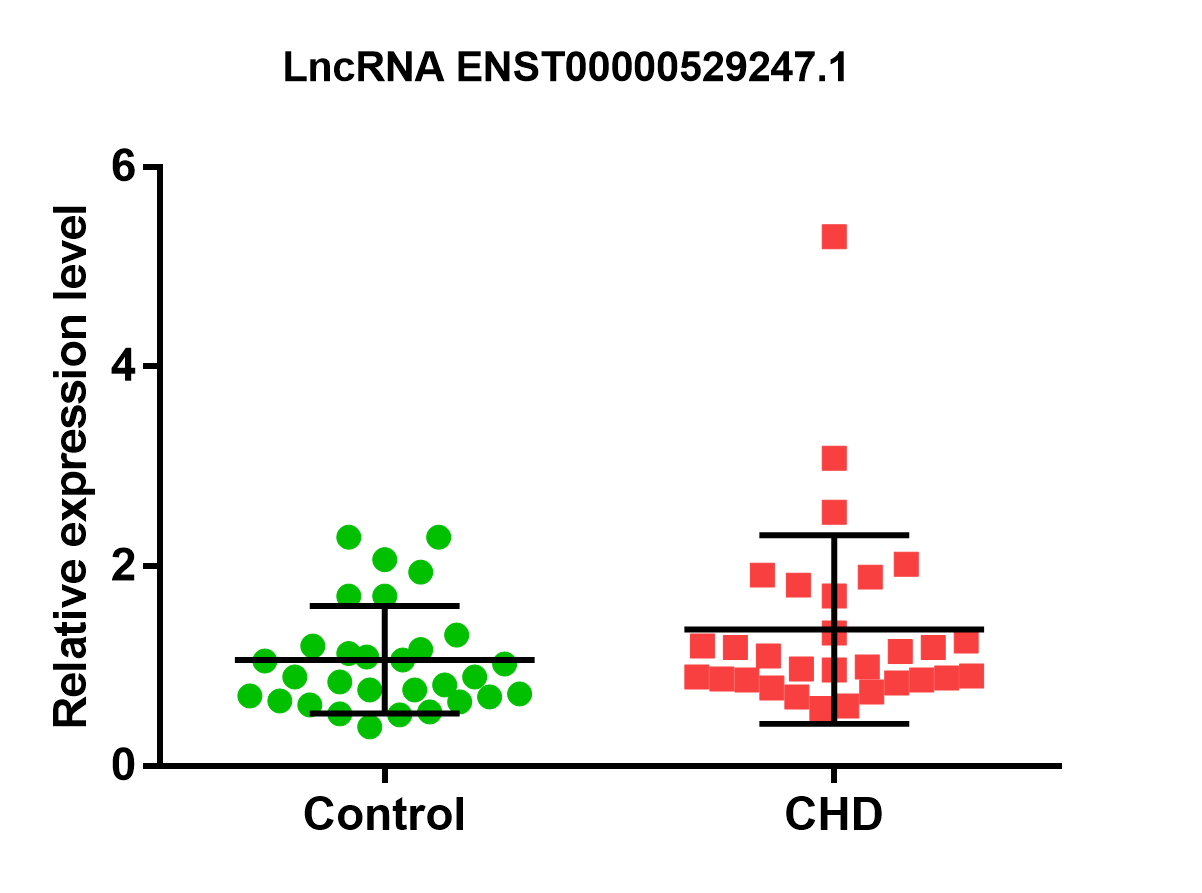

Supplement: Supplementary file 1 [file Data_Sheet_1.ZIP › raw data/Fig.2/247.1.tif]

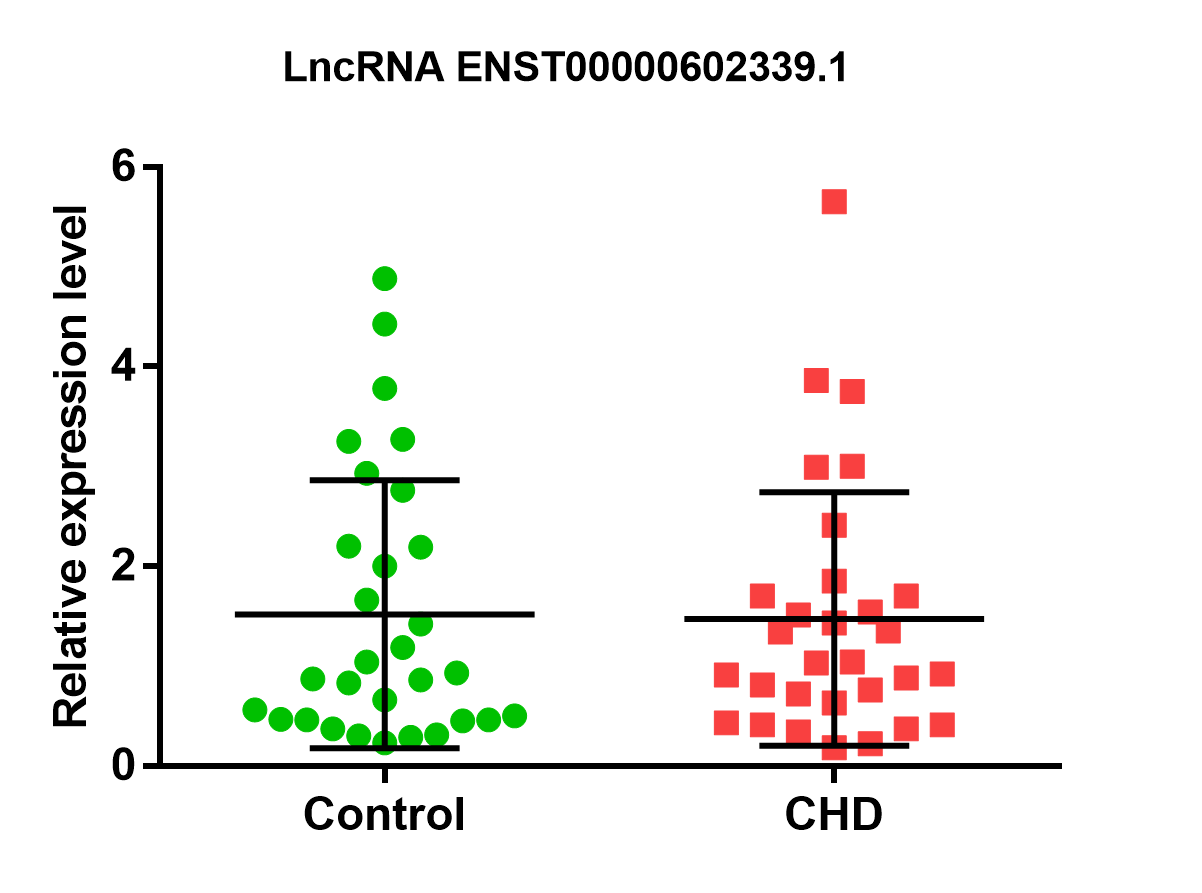

Supplement: Supplementary file 1 [file Data_Sheet_1.ZIP › raw data/Fig.2/339.1.tif]

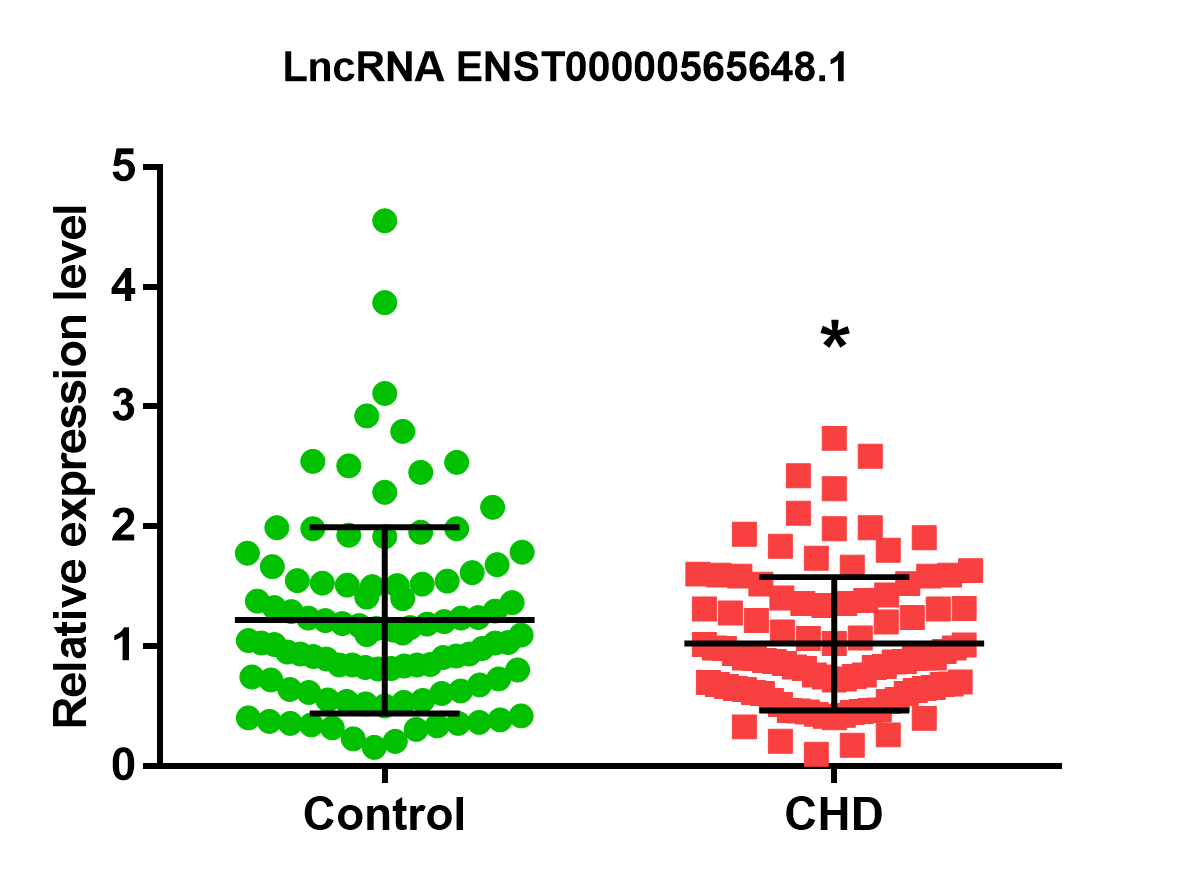

Supplement: Supplementary file 1 [file Data_Sheet_1.ZIP › raw data/Fig.2/448.1.tif]

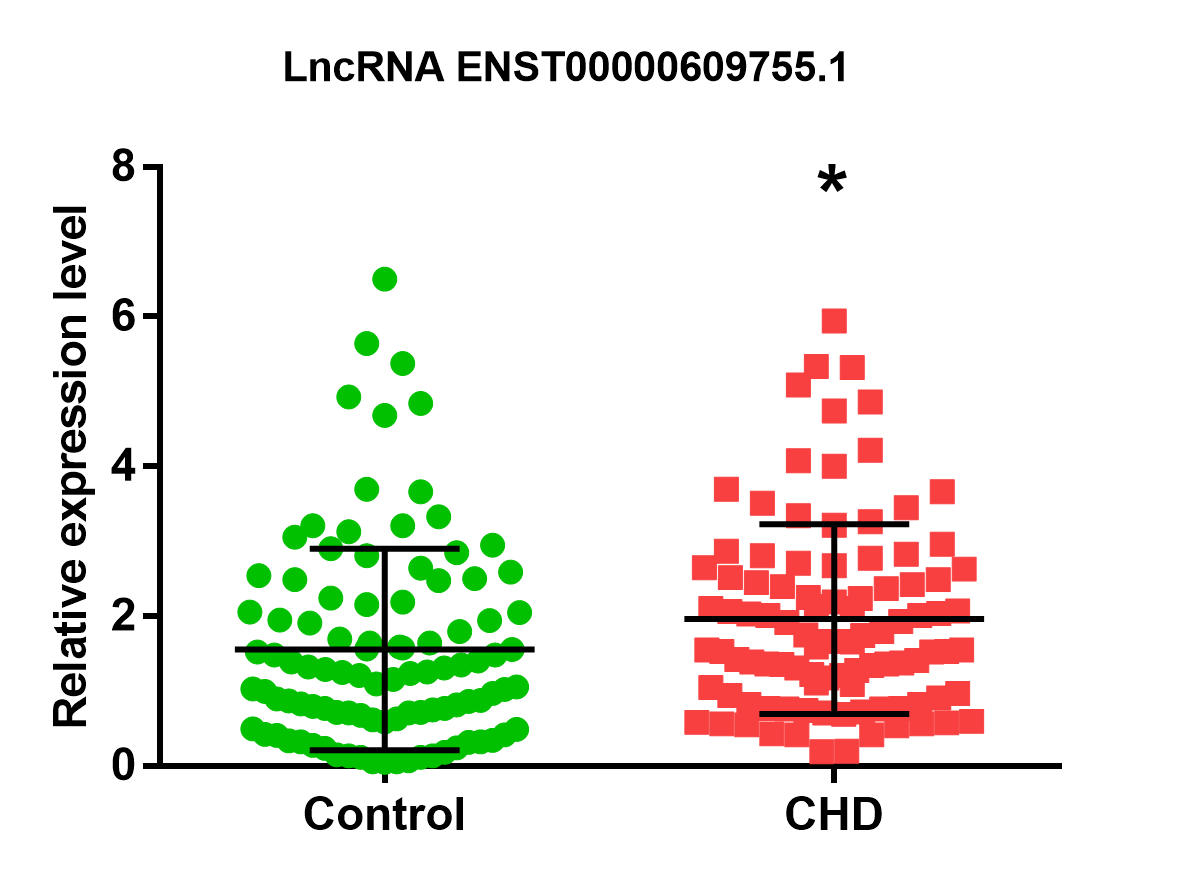

Supplement: Supplementary file 1 [file Data_Sheet_1.ZIP › raw data/Fig.2/755.1.tif]

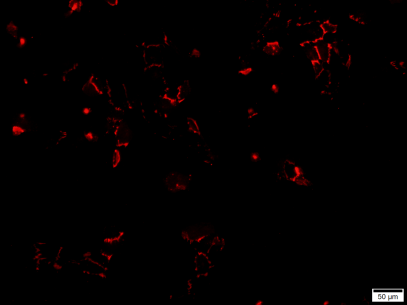

Supplement: Supplementary file 1 [file Data_Sheet_1.ZIP › raw data/Fig.3/1.tif]

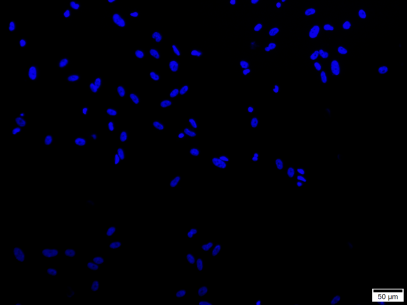

Supplement: Supplementary file 1 [file Data_Sheet_1.ZIP › raw data/Fig.3/2.tif]

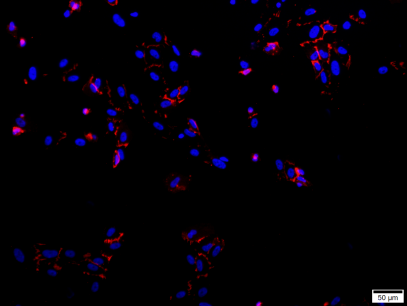

Supplement: Supplementary file 1 [file Data_Sheet_1.ZIP › raw data/Fig.3/3.tif]

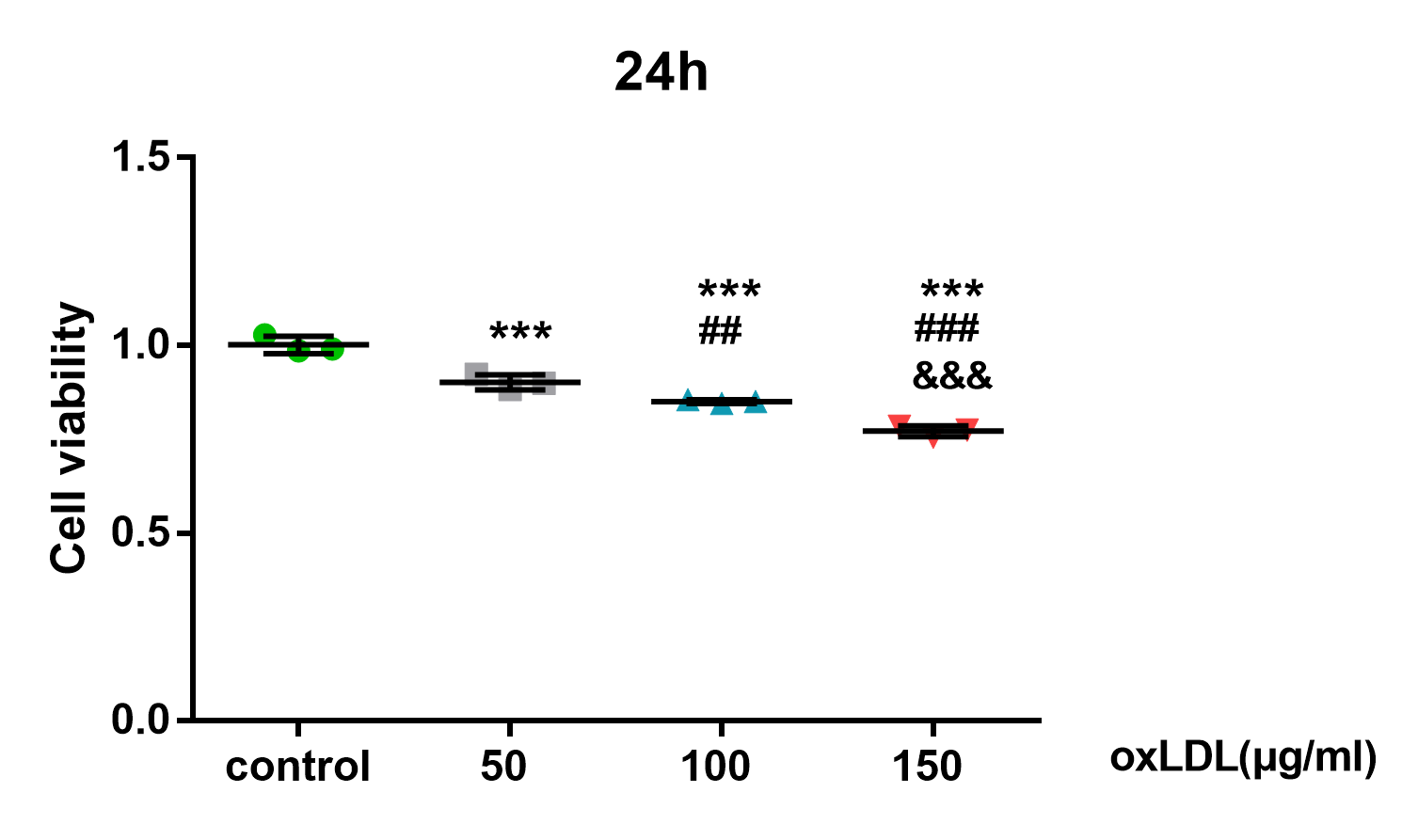

Supplement: Supplementary file 1 [file Data_Sheet_1.ZIP › raw data/Fig.3/CCK8-24h.tif]

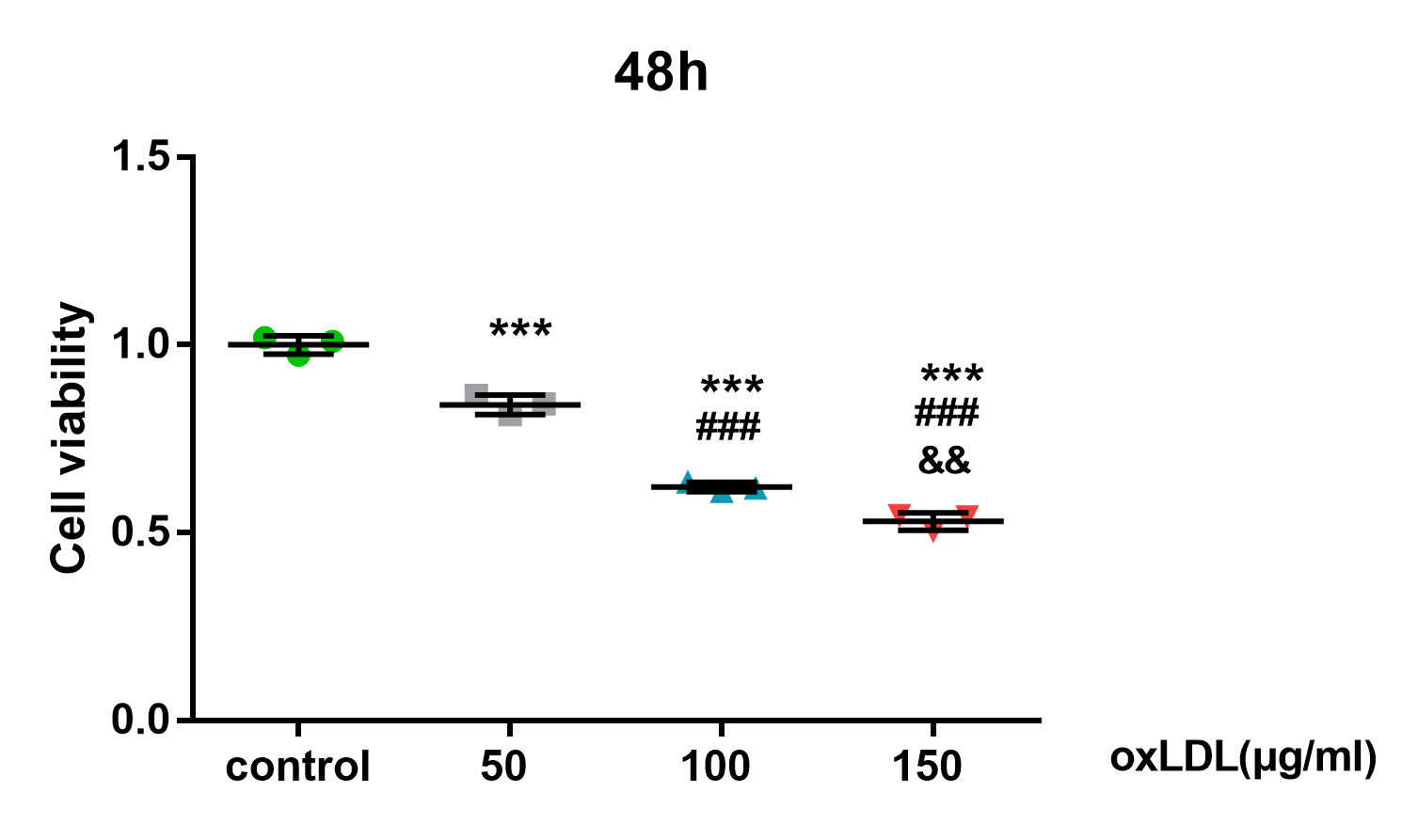

Supplement: Supplementary file 1 [file Data_Sheet_1.ZIP › raw data/Fig.3/CCK8-48h.tif]

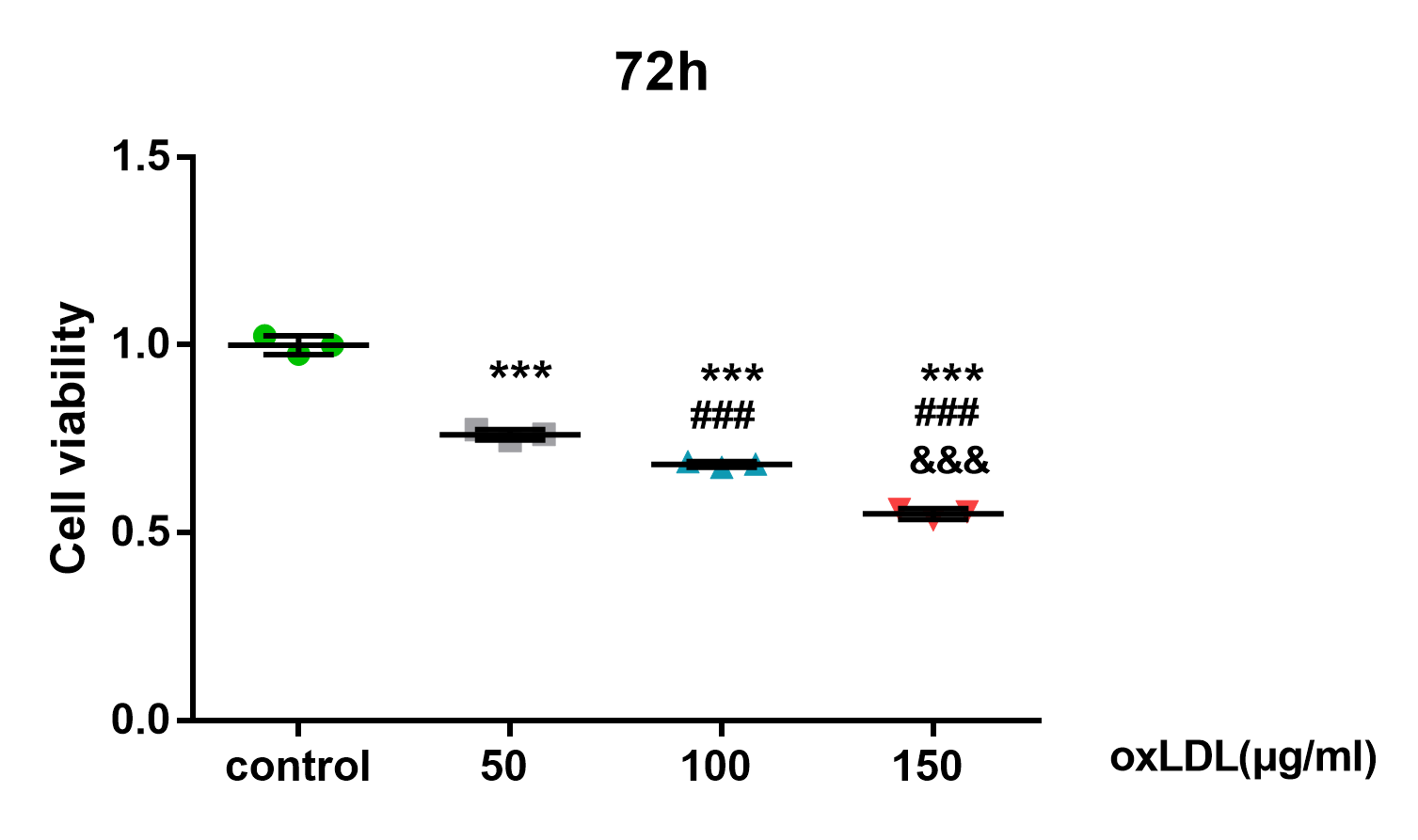

Supplement: Supplementary file 1 [file Data_Sheet_1.ZIP › raw data/Fig.3/CCK8-72h.tif]

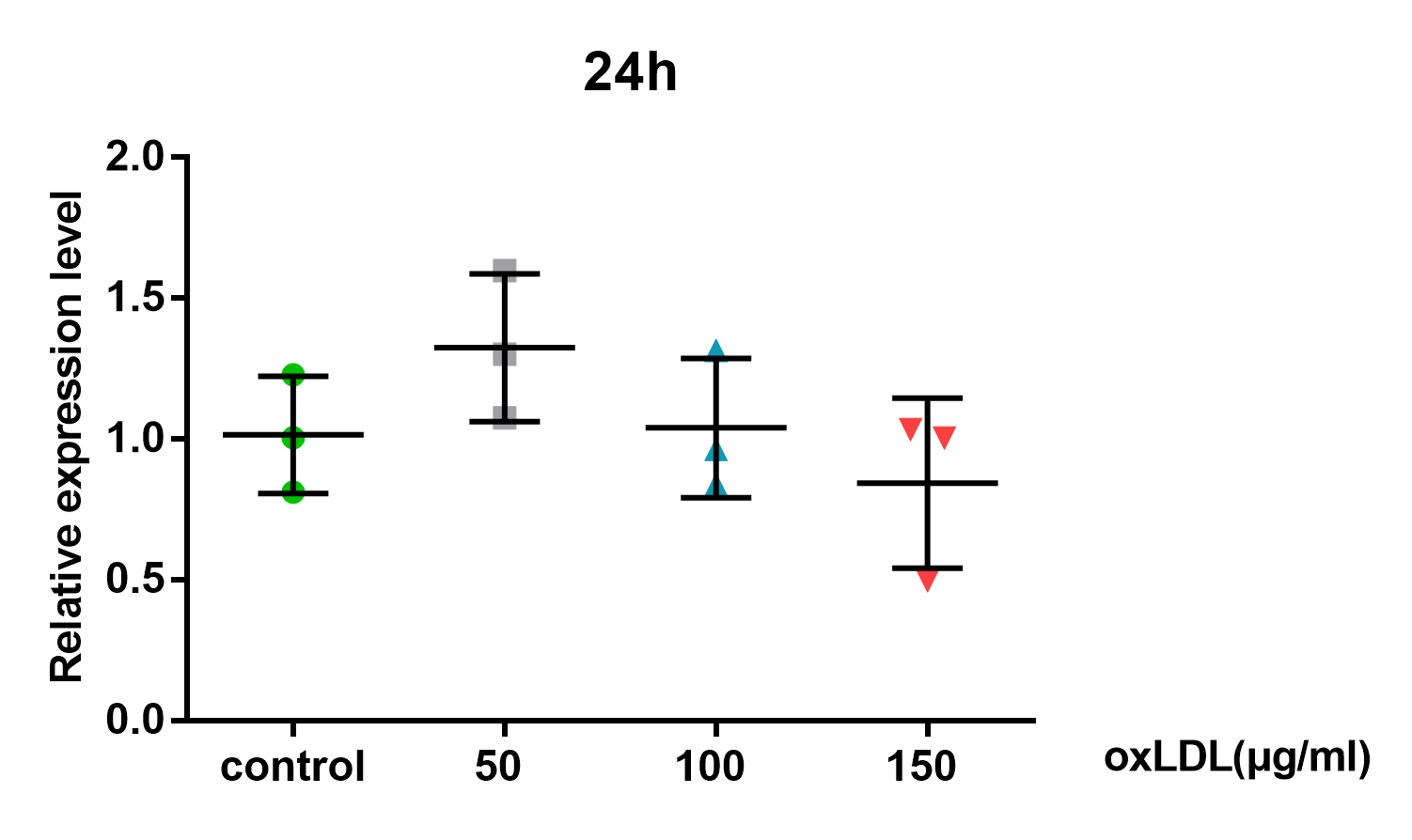

Supplement: Supplementary file 1 [file Data_Sheet_1.ZIP › raw data/Fig.3/cell-48.1-24h.tif]

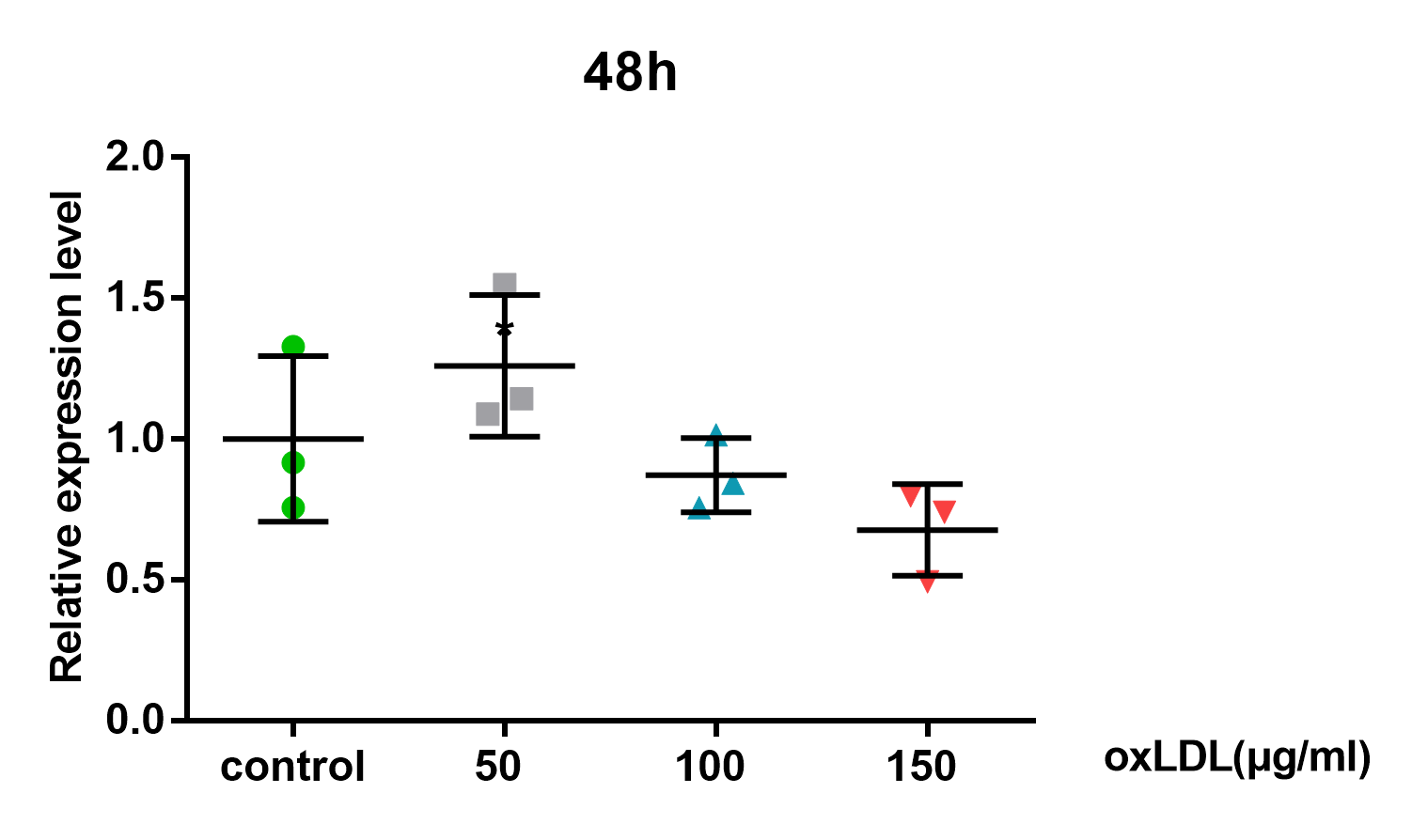

Supplement: Supplementary file 1 [file Data_Sheet_1.ZIP › raw data/Fig.3/cell-48.1-48h.tif]

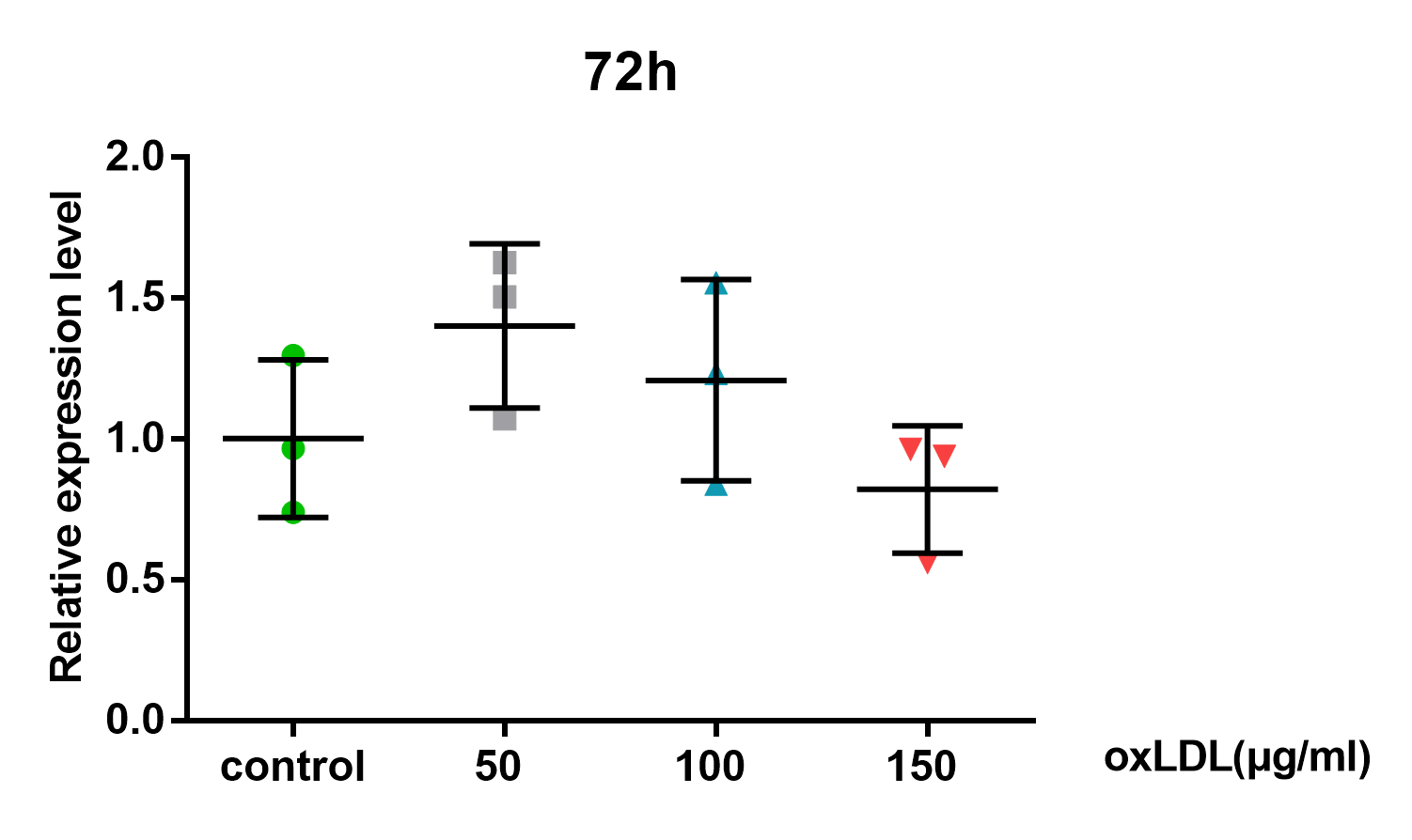

Supplement: Supplementary file 1 [file Data_Sheet_1.ZIP › raw data/Fig.3/cell-48.1-72h.tif]

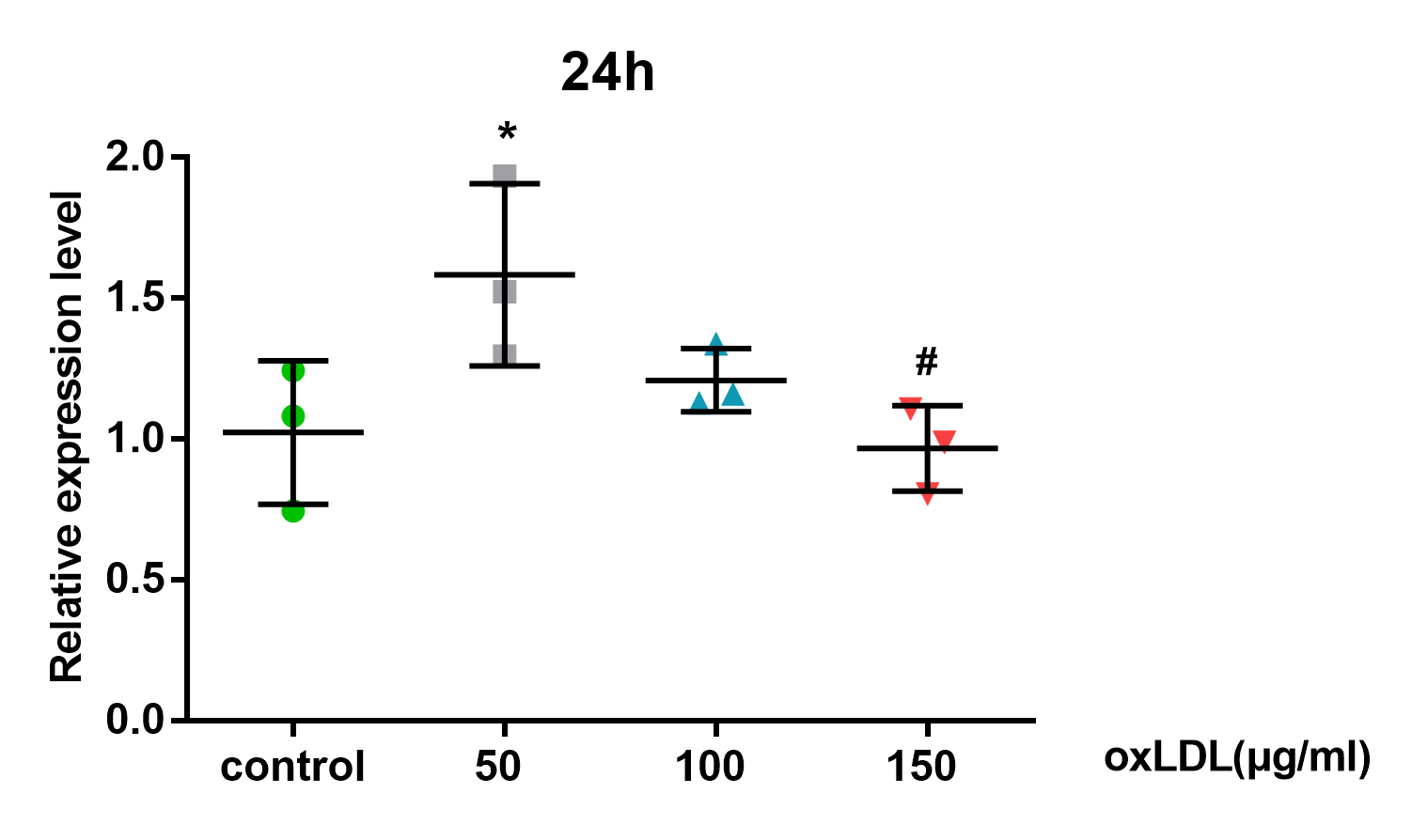

Supplement: Supplementary file 1 [file Data_Sheet_1.ZIP › raw data/Fig.3/cell-755.1-24h.tif]

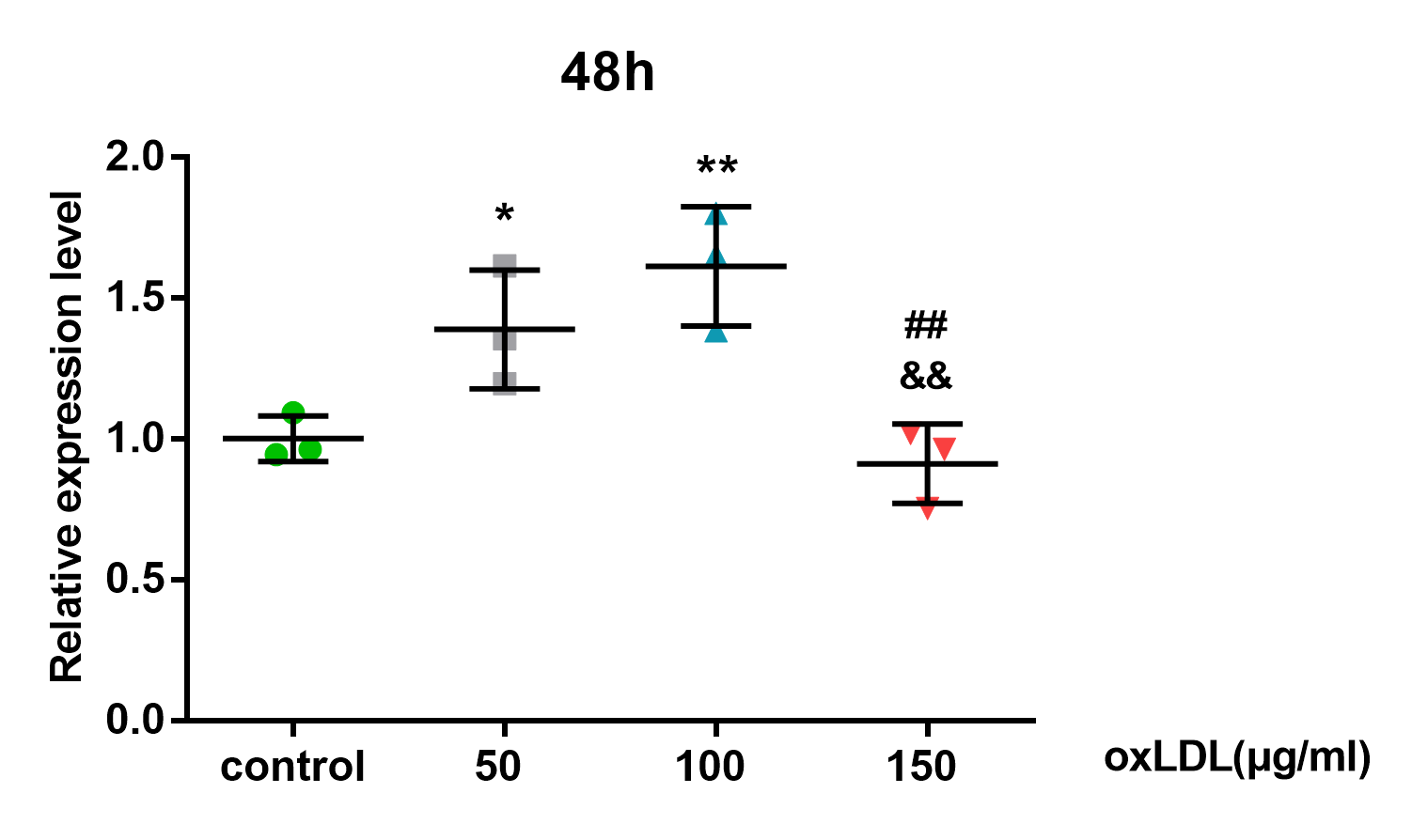

Supplement: Supplementary file 1 [file Data_Sheet_1.ZIP › raw data/Fig.3/cell-755.1-48h.tif]

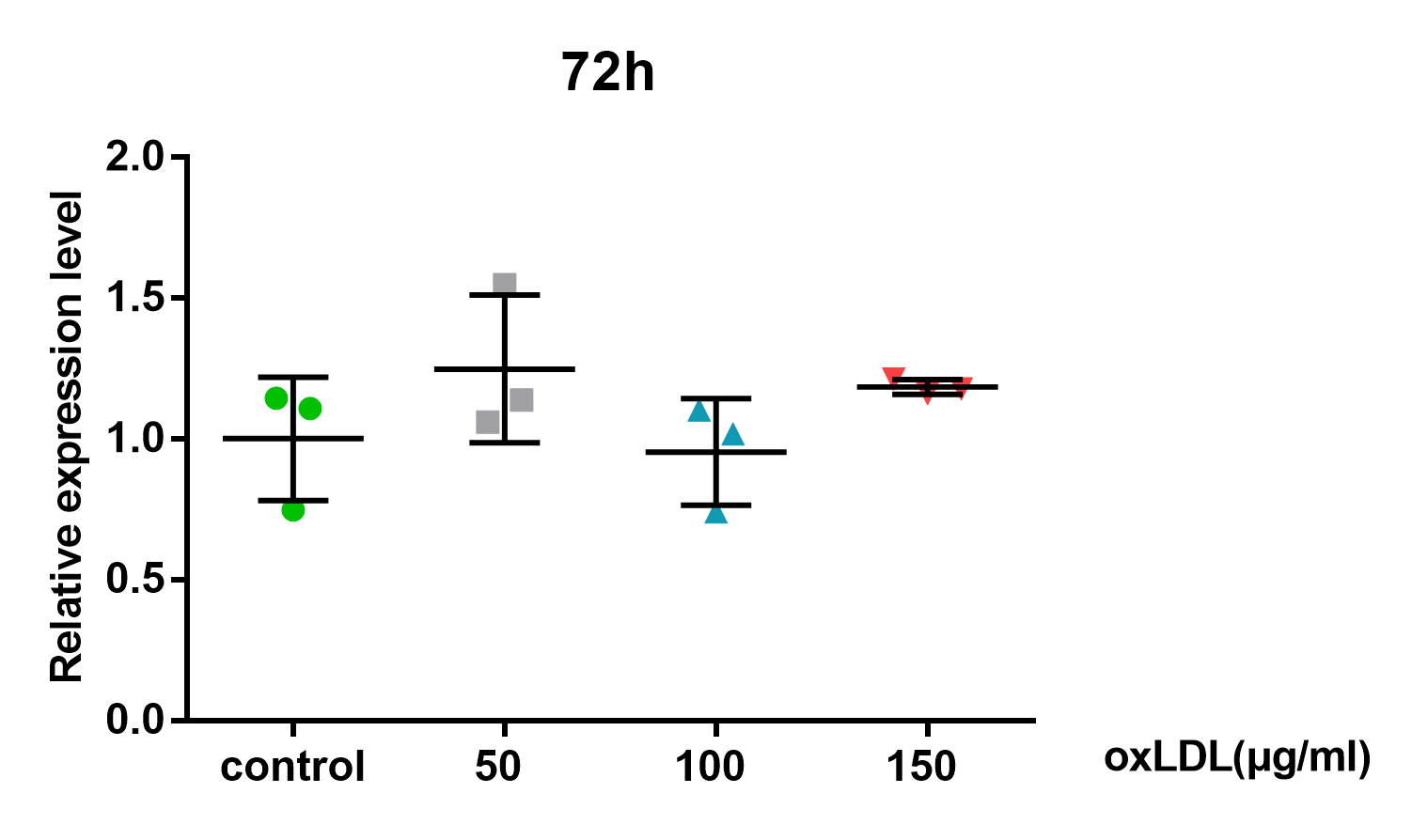

Supplement: Supplementary file 1 [file Data_Sheet_1.ZIP › raw data/Fig.3/cell-755.1-72h.tif]

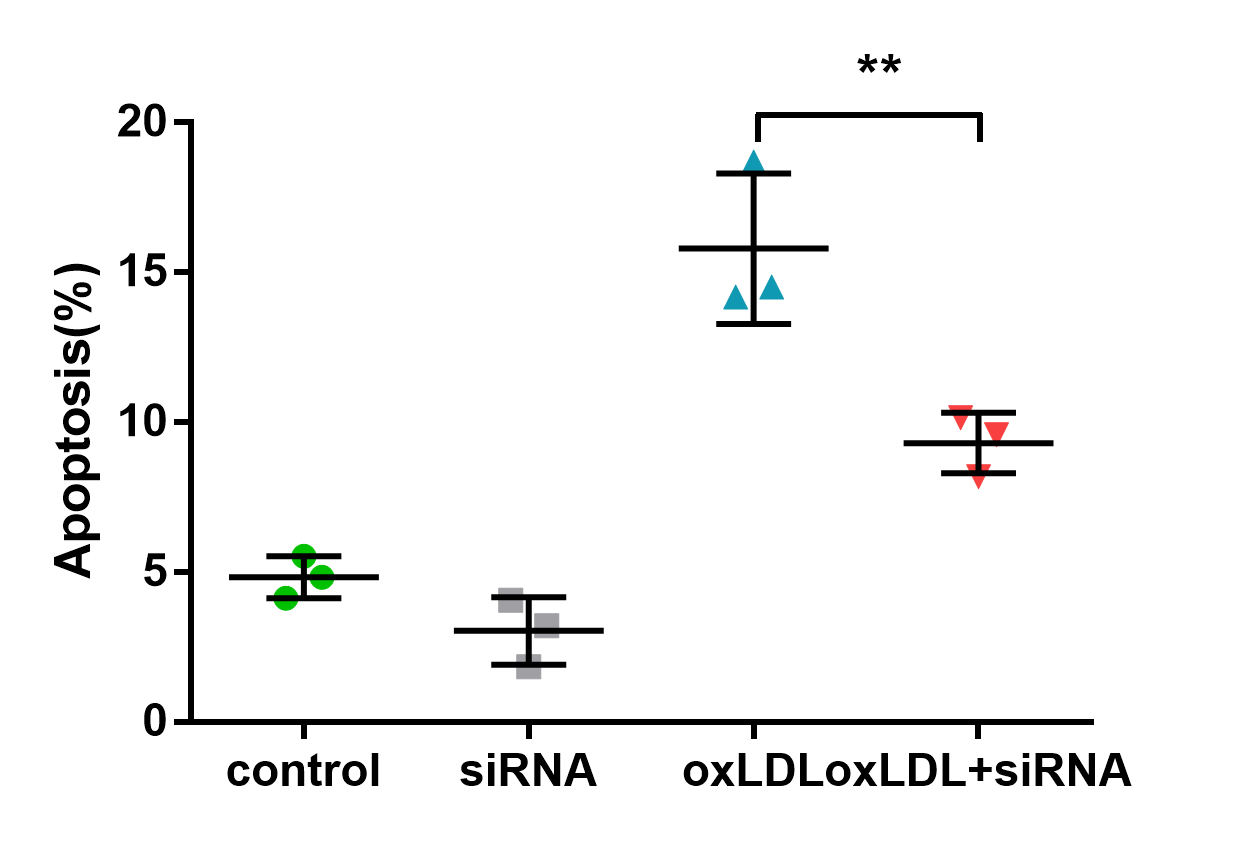

Supplement: Supplementary file 1 [file Data_Sheet_1.ZIP › raw data/Fig.4/-siRNA-oxLDL(g).tif]

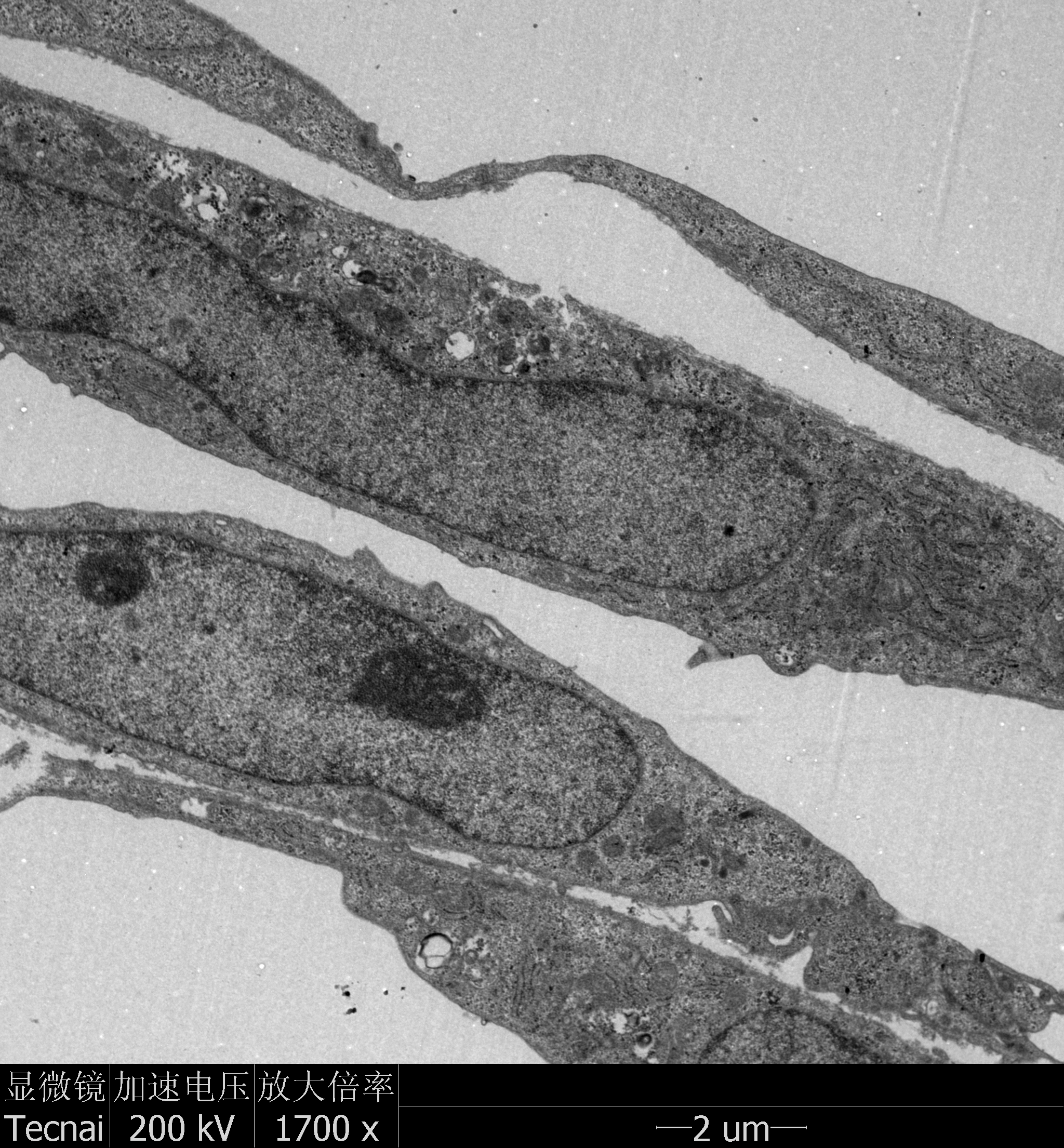

Supplement: Supplementary file 1 [file Data_Sheet_1.ZIP › raw data/Fig.4/1 (1).tif]

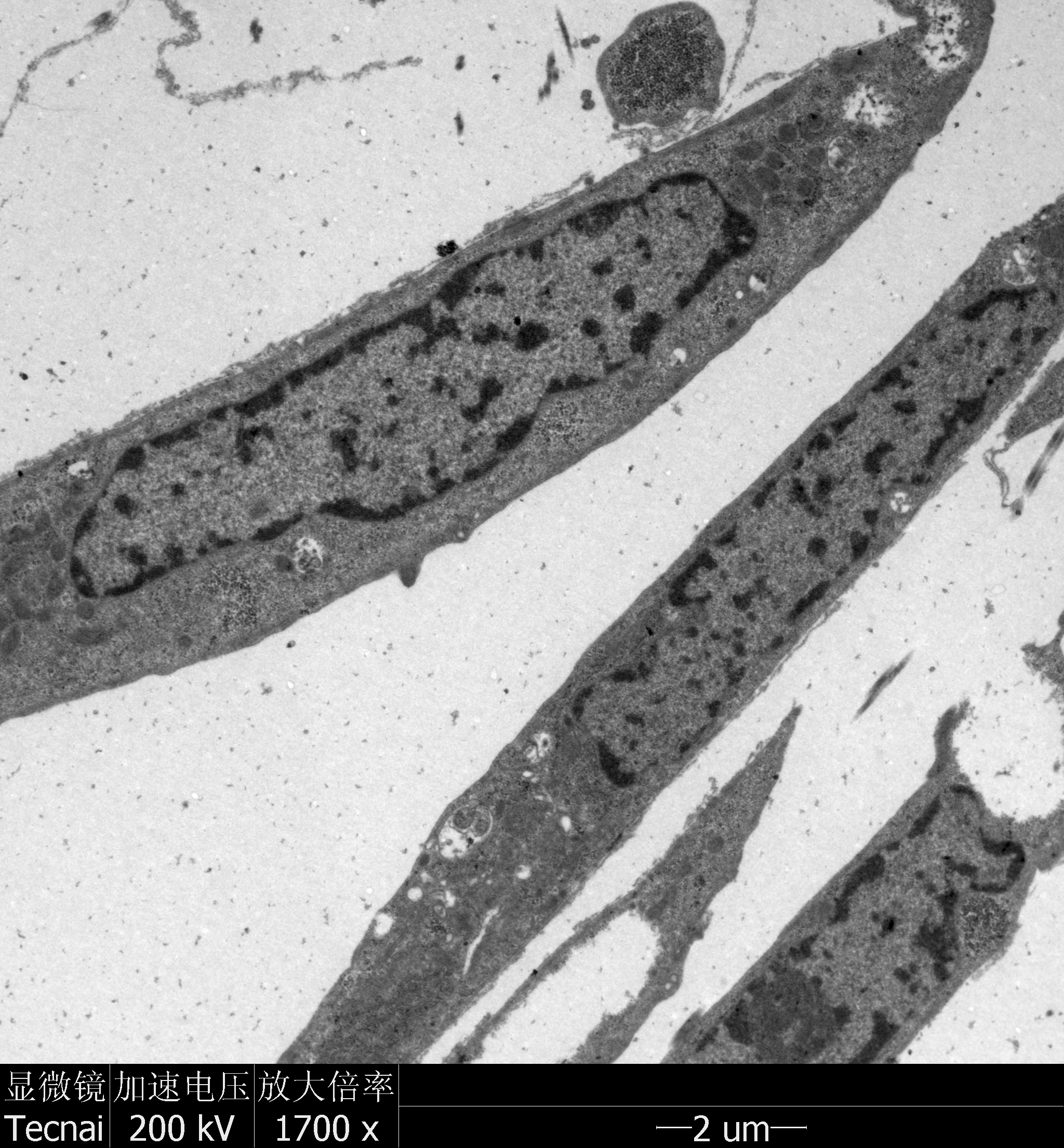

Supplement: Supplementary file 1 [file Data_Sheet_1.ZIP › raw data/Fig.4/1 (13).tif]

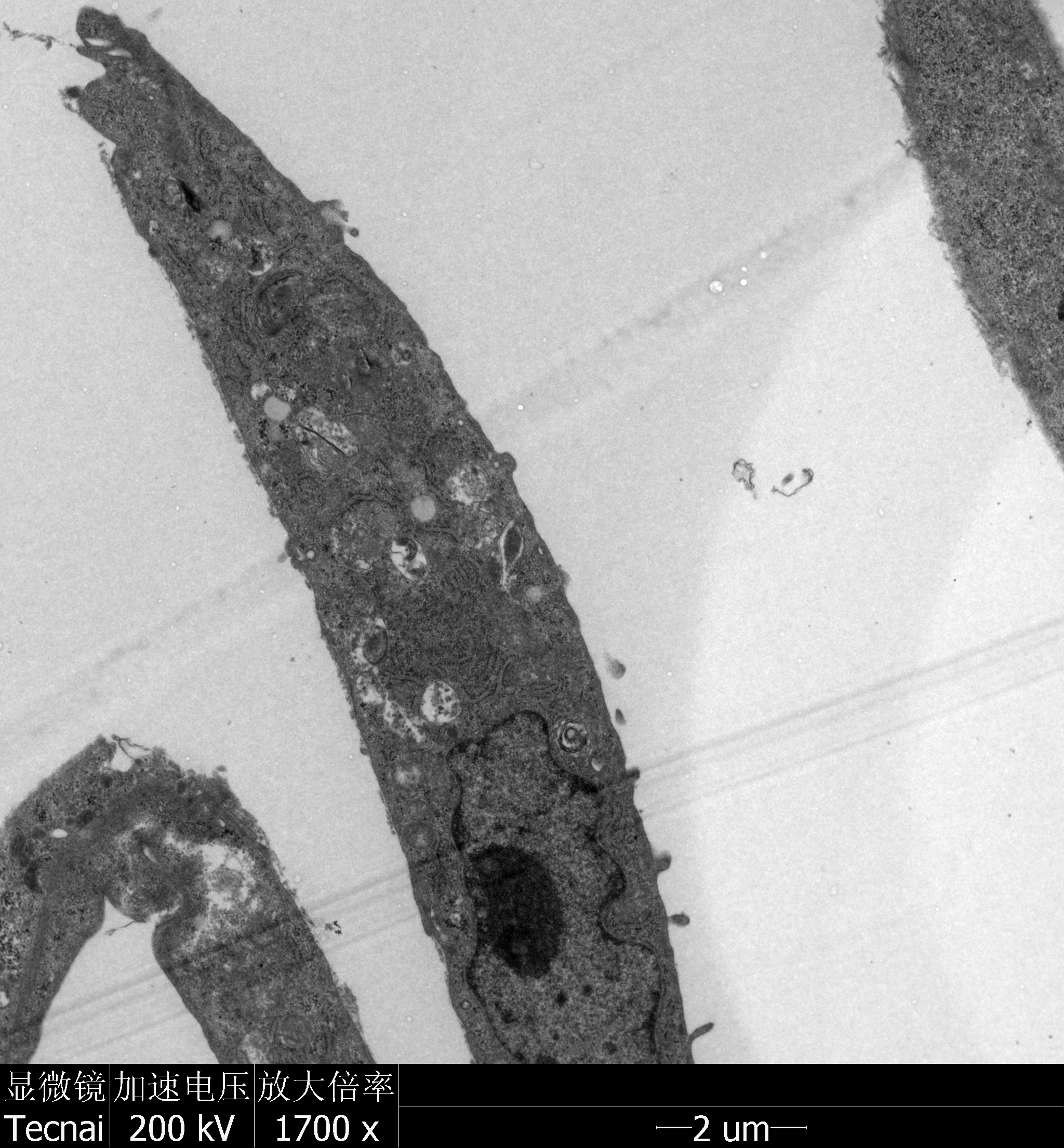

Supplement: Supplementary file 1 [file Data_Sheet_1.ZIP › raw data/Fig.4/1 (14).tif]

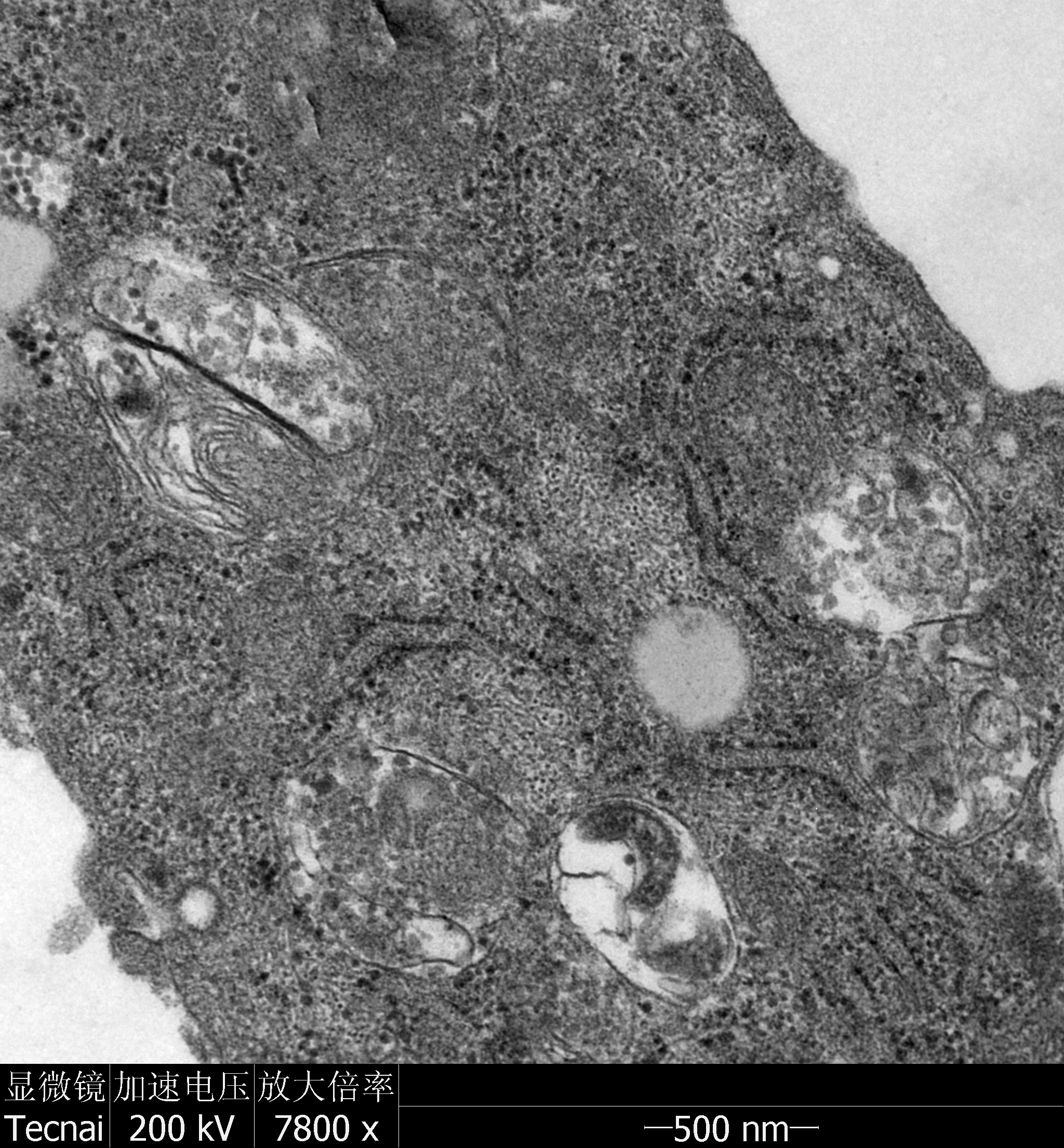

Supplement: Supplementary file 1 [file Data_Sheet_1.ZIP › raw data/Fig.4/1 (15).tif]

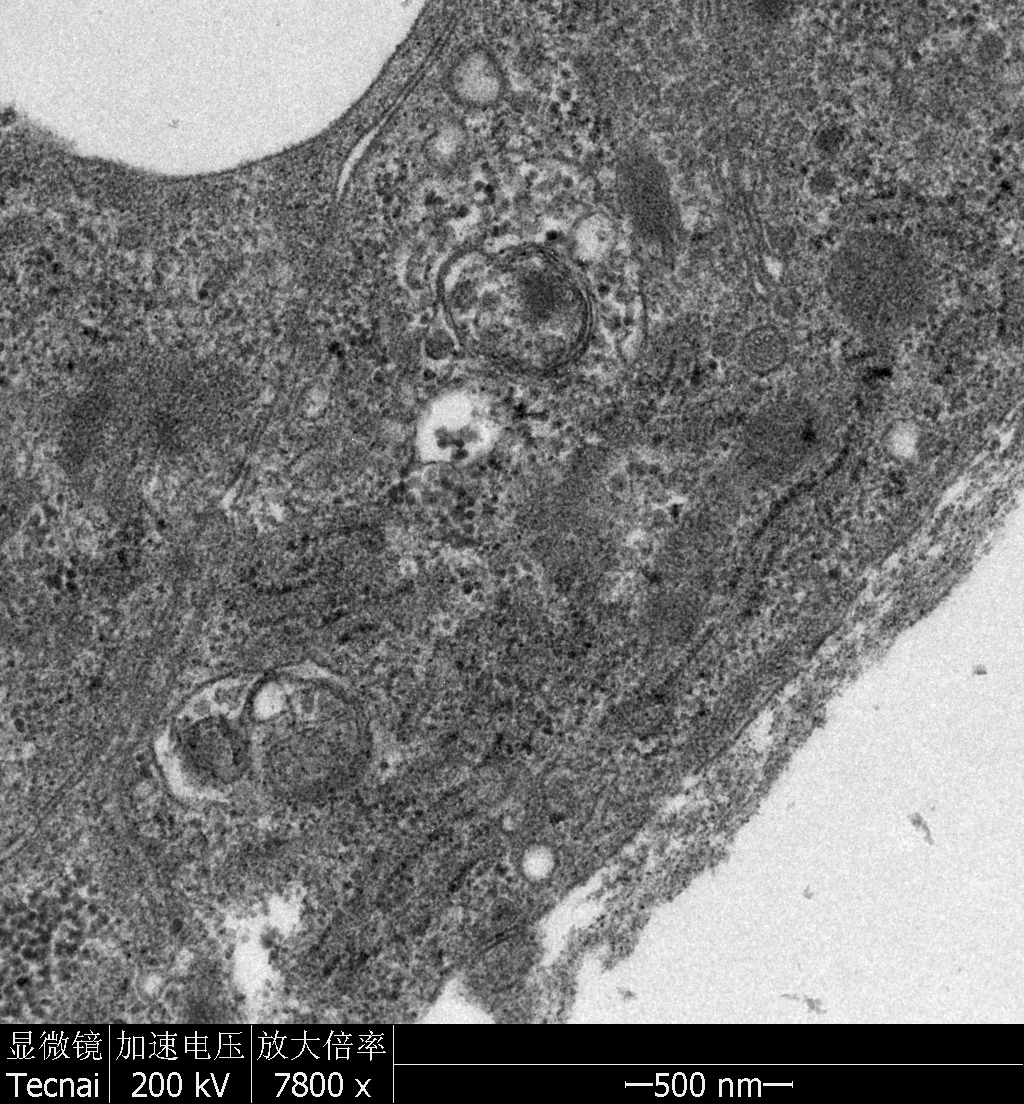

Supplement: Supplementary file 1 [file Data_Sheet_1.ZIP › raw data/Fig.4/1 (3).tif]

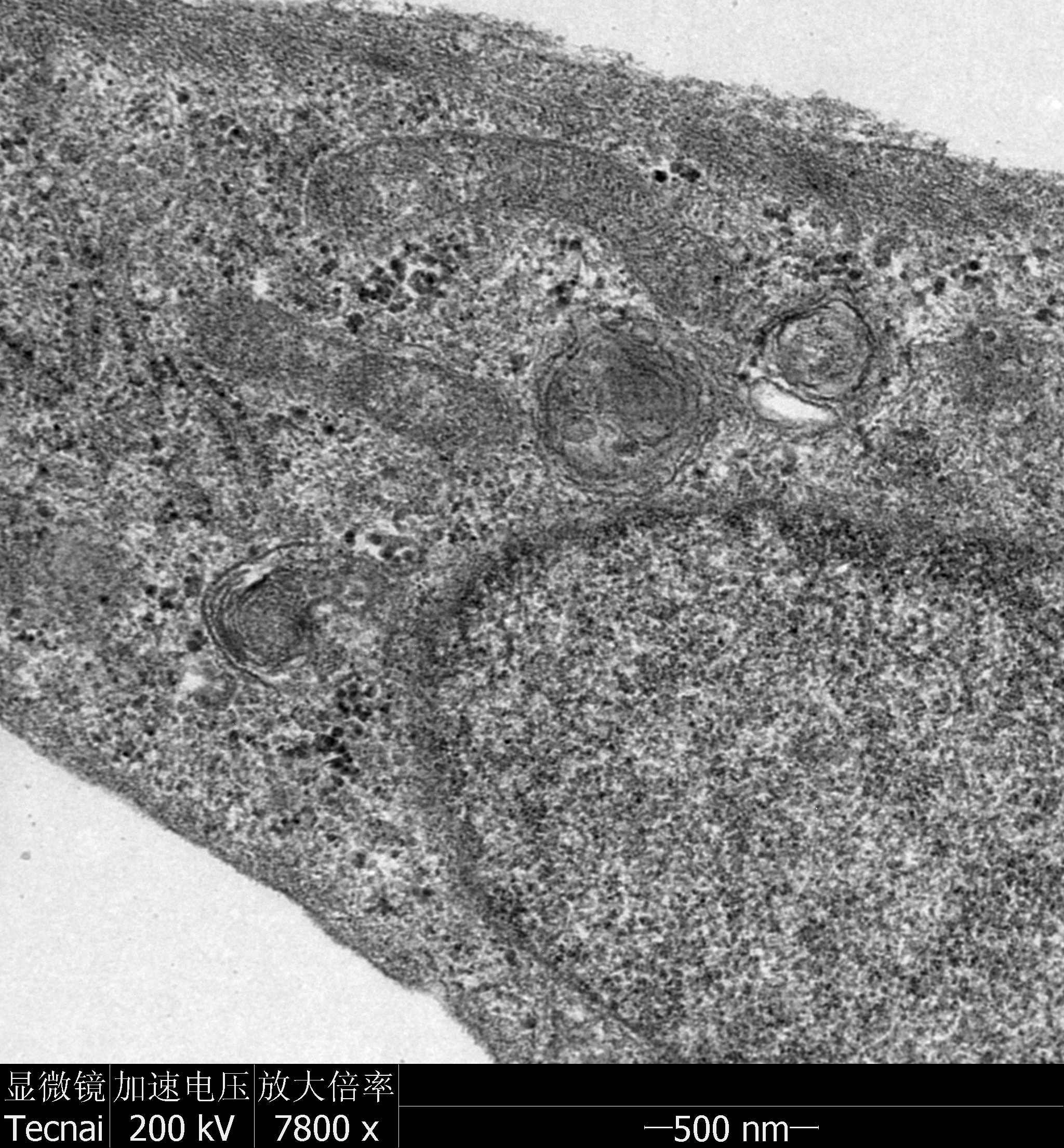

Supplement: Supplementary file 1 [file Data_Sheet_1.ZIP › raw data/Fig.4/1 (6).tif]

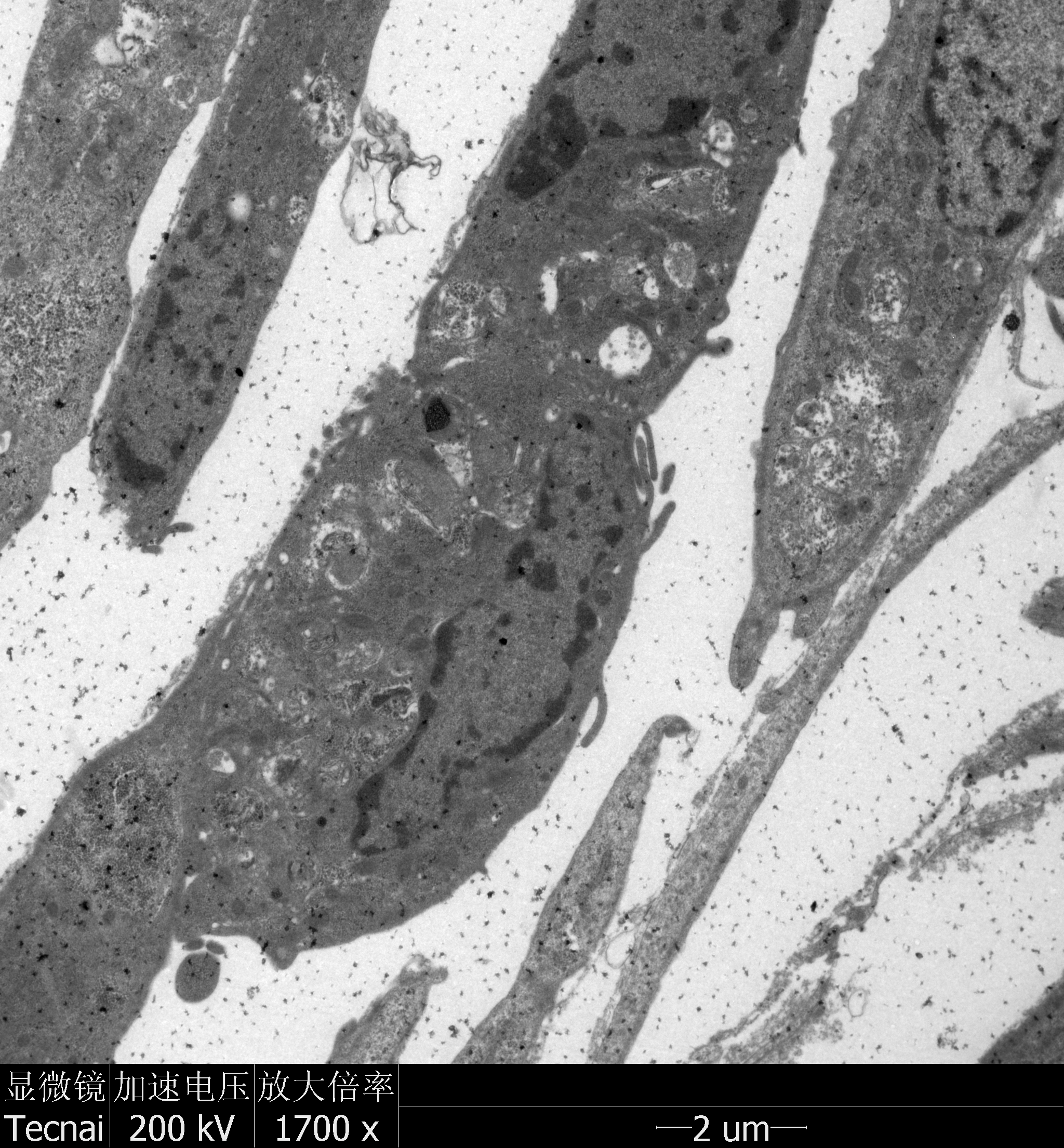

Supplement: Supplementary file 1 [file Data_Sheet_1.ZIP › raw data/Fig.4/2 (8).tif]

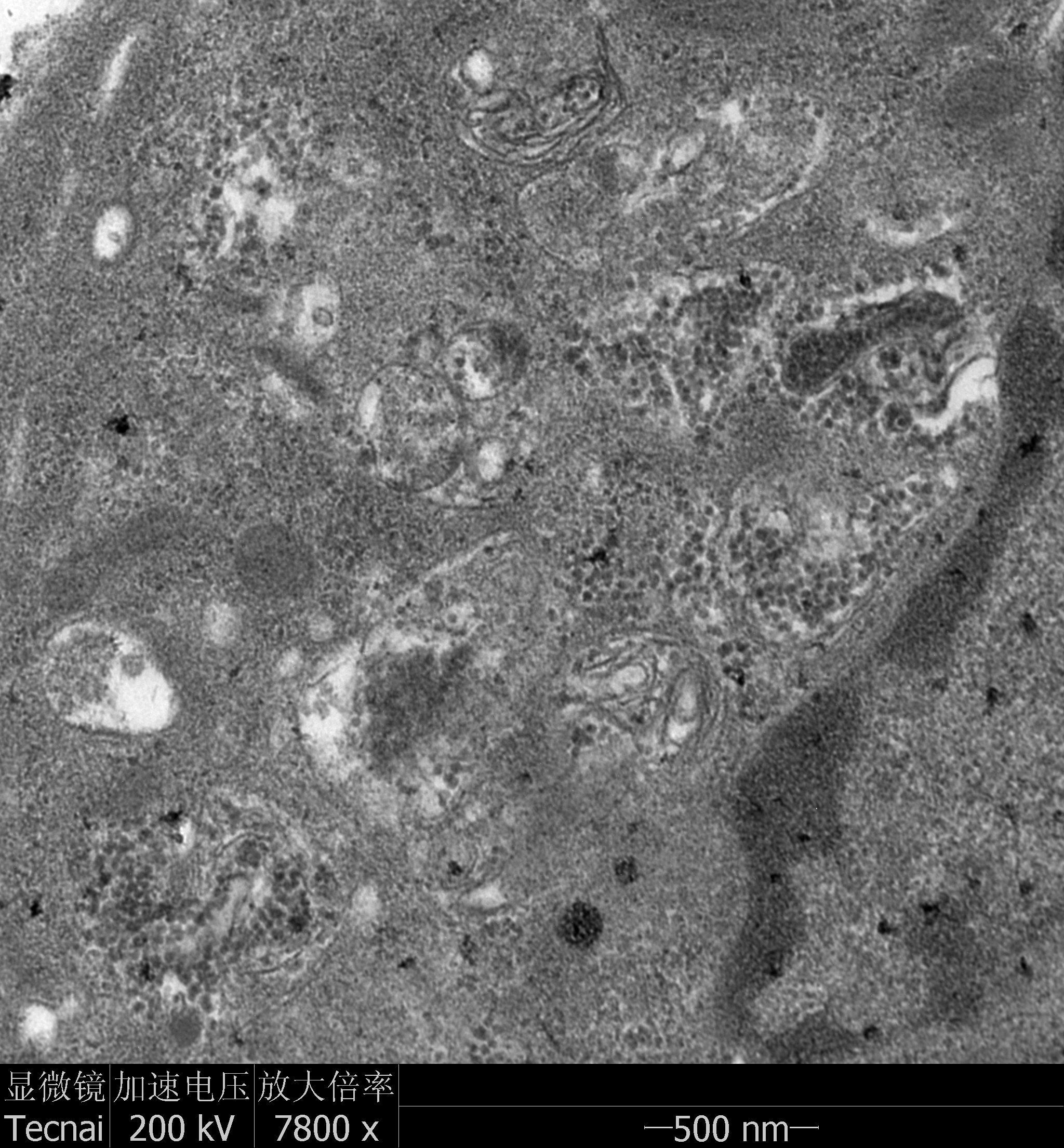

Supplement: Supplementary file 1 [file Data_Sheet_1.ZIP › raw data/Fig.4/2 (9).tif]

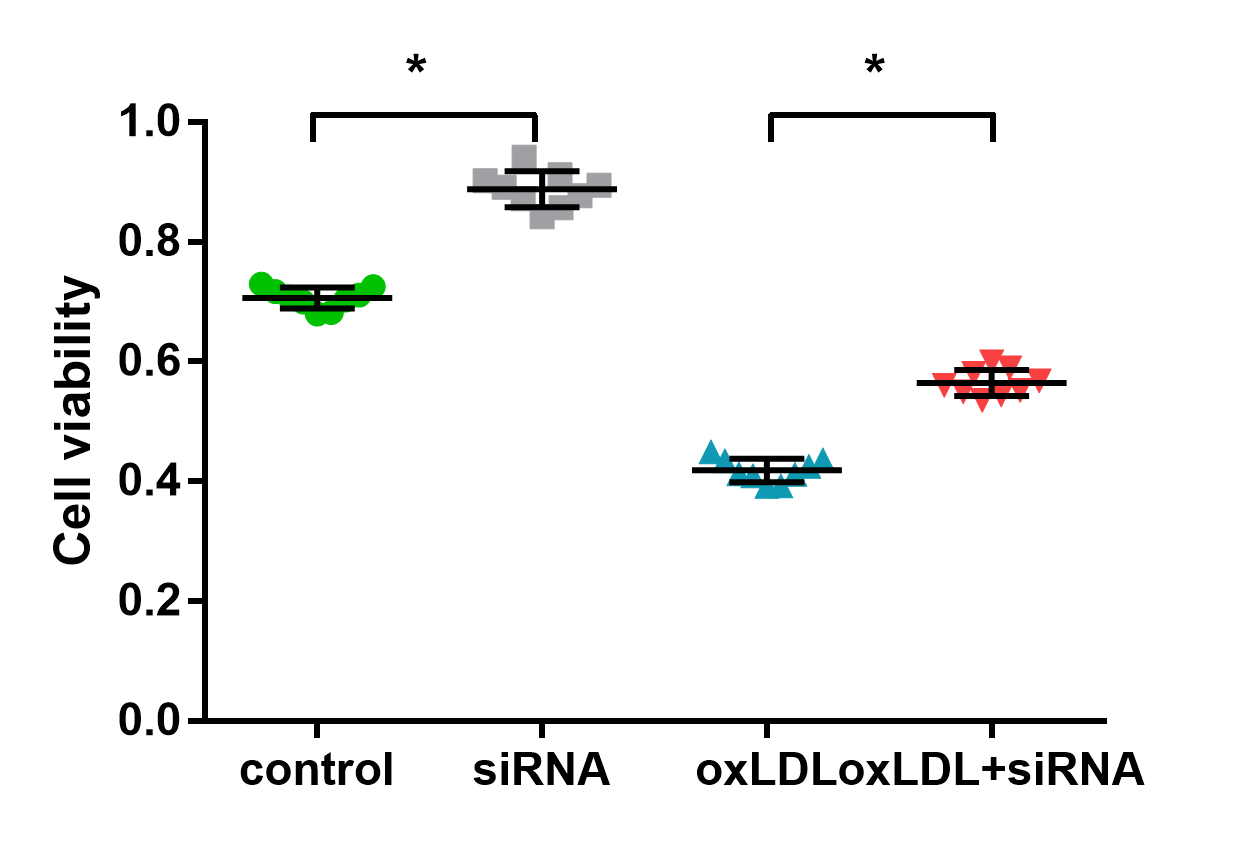

Supplement: Supplementary file 1 [file Data_Sheet_1.ZIP › raw data/Fig.4/CCK8-siRNA-oxLDL(e).tif]

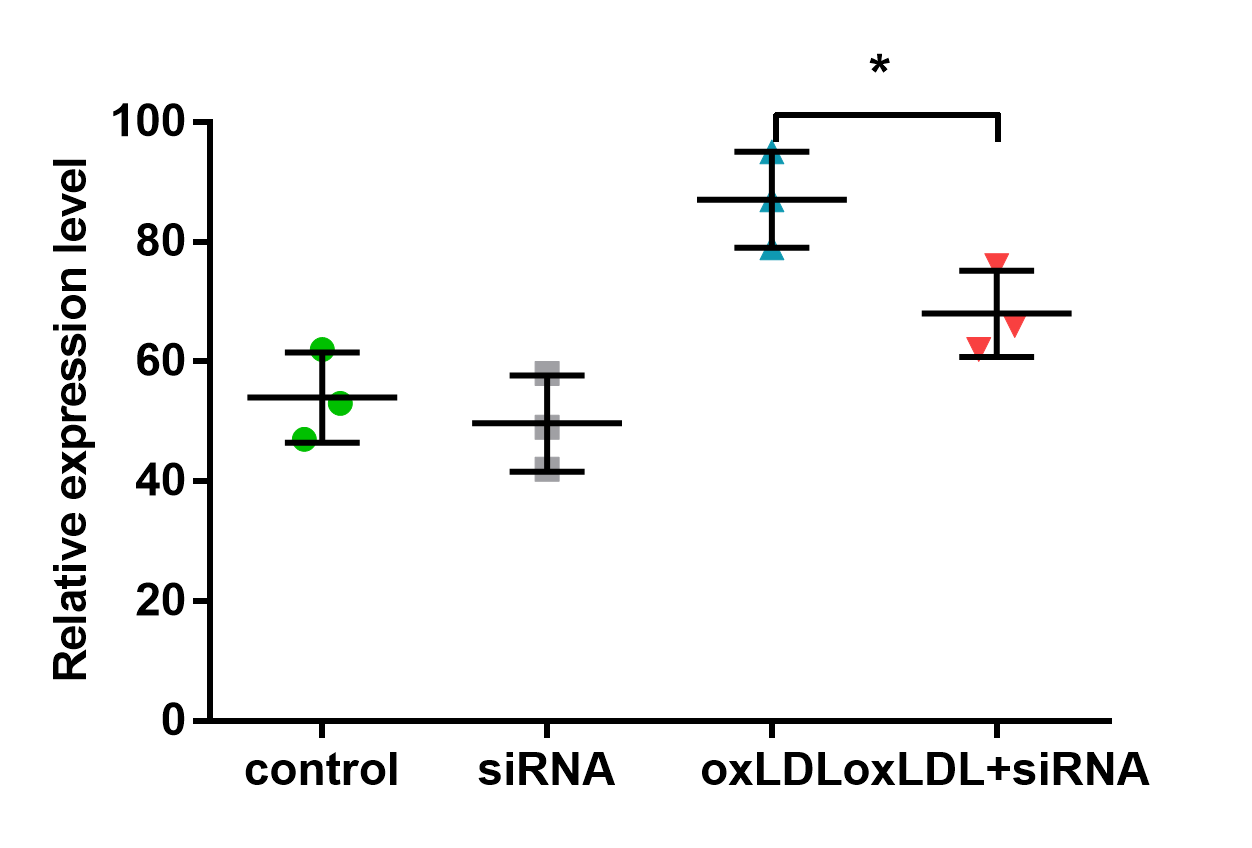

Supplement: Supplementary file 1 [file Data_Sheet_1.ZIP › raw data/Fig.4/LDH-siRNA-oxLDL(d).tif]

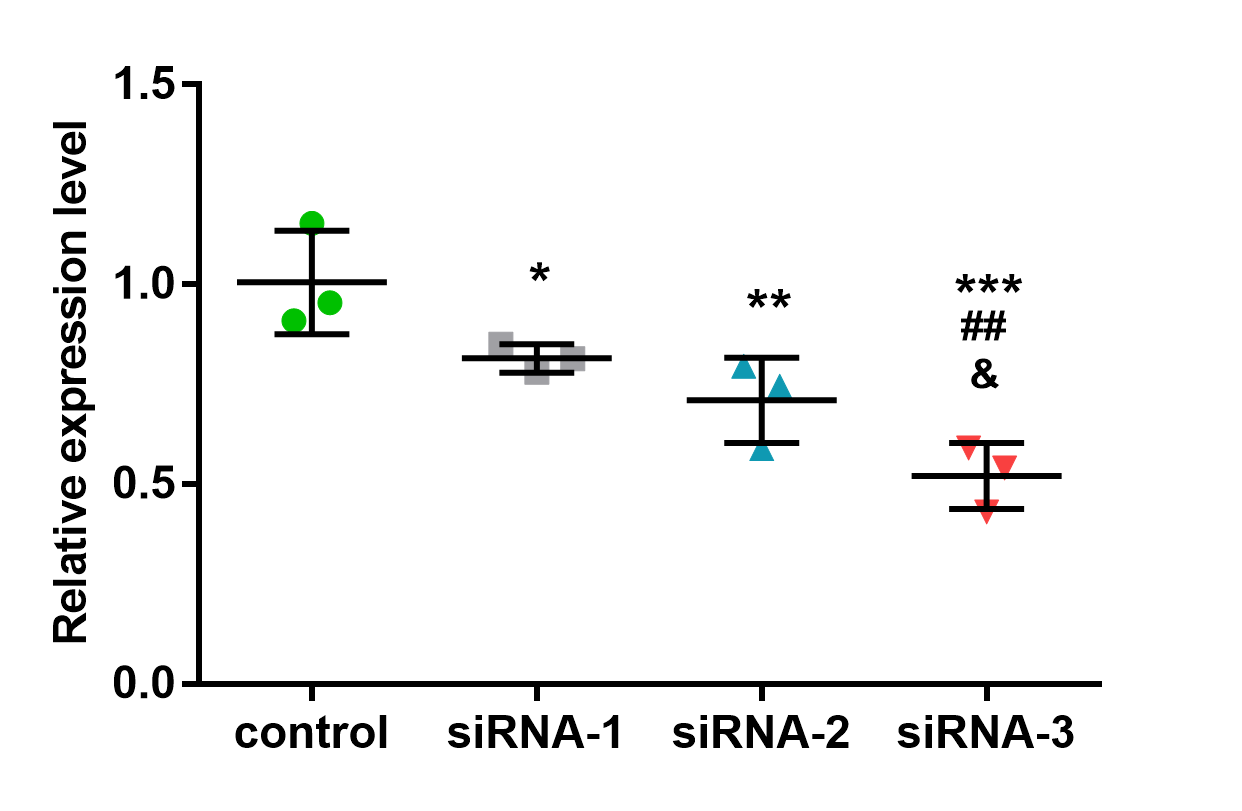

Supplement: Supplementary file 1 [file Data_Sheet_1.ZIP › raw data/Fig.4/siRNA (a).tif]

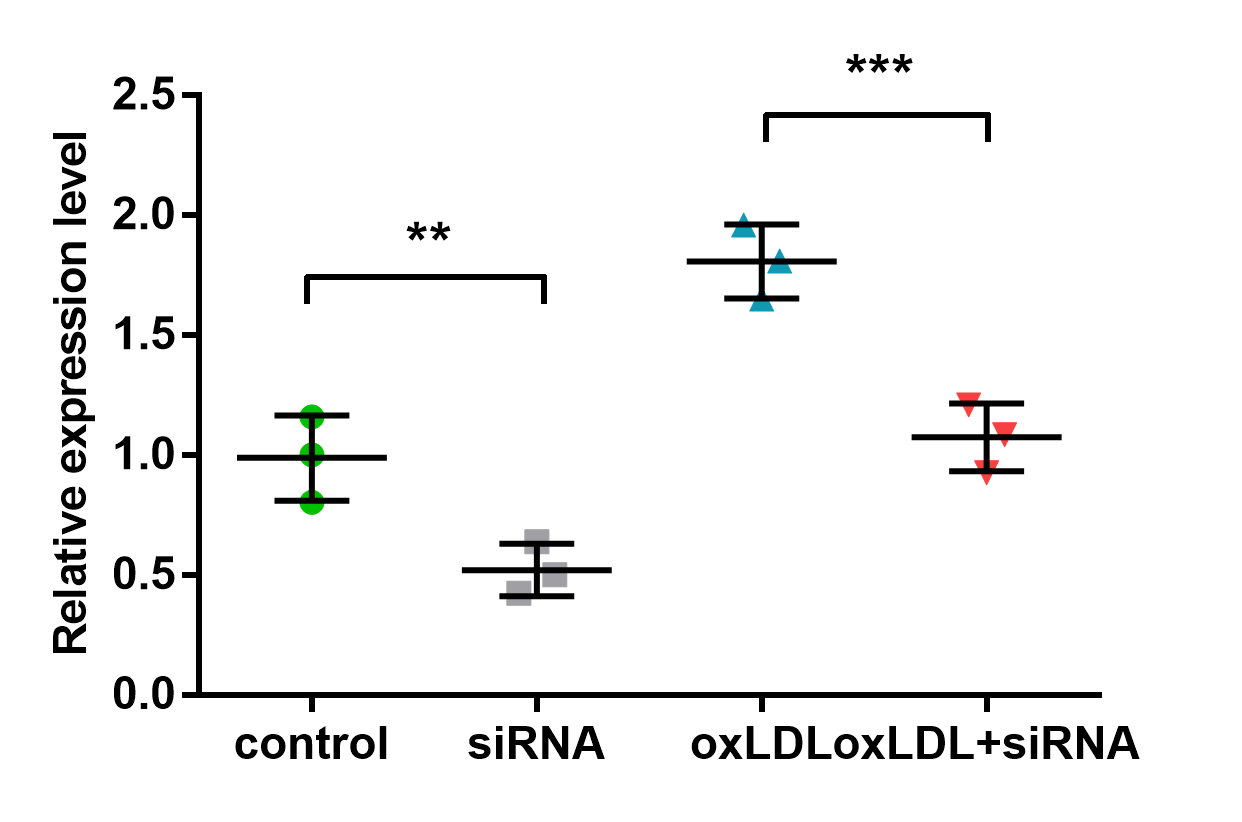

Supplement: Supplementary file 1 [file Data_Sheet_1.ZIP › raw data/Fig.4/siRNA-oxLDL(b).tif]
