# Supplementary material for: Knockdown of lncRNA ENST00000609755.1 Confers Protection Against Early oxLDL-Induced Coronary Heart Disease
Source: Front Cardiovasc Med. 2021 May 21;8:650212. doi: 10.3389/fcvm.2021.650212 (PMC8175657; doi:10.3389/fcvm.2021.650212)
Supplement: Supplementary file 2 [file Data_Sheet_2.doc]

**Supplementary Methods**

**Definition of environmental risk factors of CHD**

Family history of CHD was defined if one of the participant’s biological parents or siblings had developed CHD in any site. Tobacco smokers comprised those who had smoked at least 100 tobacco cigarettes during their lifetime. Alcohol drinking was considered when the patients had continuously consumed at least 1 drink per week for at least 6 months. Tea drinkers included subjects who continuously consumed at least one cup of tea per week for at least 6 months. A high-salt diet means eating more than 6 grams of salt per day. Labor intensity was classified into three categories depending on intensity, duration, and frequency of the participant’s physical activity at work as follows: light-intensity, 75% of the time sitting or standing and 25% of the time for standing activities, such as office work, repair electrical appliances and clocks, salesmen, hotel attendants, etc; moderate-intensity, 25% of the time sitting or standing and 75% of the time participating in special activities, such as motor vehicle driving, electrician installation and lathe operation; and high-intensity work, 40% of the time for sitting or standing and 60% of the time for special occupation activities, such as Agricultural labor, steelmaking, dance, sports activities, handling, mining, etc [1]. As for "self-rating anxiety scale (SAS)" and "self-rating depression scale (SDS)". The SAS was designed by William WK Zung in 1971 to quantify a patient’ s level of anxiety. The SAS scale is a 20-item self-reported assessment device. When answering each item, the person indicates the degree to which each statement applies. Each question is scored on a Likert-type scale of 1 to 4 (based on the following replies: “a little of the time”, “some of the time”, “a good part of the time” and “most of the time”). The total score is obtained by summing the assessment of the 20 items. The total score multiplied by 1.25 gives the standard score. A standard cutoff score of 50 is usually used to diagnose anxiety [2]. The SDS contains 20 items and its design was based on the diagnostic criteria for depression. Subjects rate each item with regard to how they have felt during the past several days using a 4-point Likert scale. The raw sum score of the SDS ranges from 20 to 80 but results are usually presented as the SDS Index, which is obtained by expressing the raw score is converted to 100 points scale [3]. In brief, SAS was classified as follows: normal (<50), mild anxiety (50–59), moderate anxiety (60–69), and severe anxiety (≥70). The SDS was classified as follows: normal (<53), mild anxiety (53–62), moderate anxiety (63–72), and severe anxiety (≥73). The recorded body mass index (BMI) was calculated as follows: BMI=weight (kg) / [height (m)]^2^ (underweight, <18.5; normal weight, 18.5–24; overweight or obesity, ≥24.

[1] Zhao W, Cong L. [Physical activity evaluation: metabolic equivalent intensity levels and evaluation of different physical activity]. Wei Sheng Yan Jiu. 2004 Mar;33(2):246-9.

[2] Zung WW, Magruder-Habib K, Velez R, Alling W. The comorbidity of anxiety and depression in general medical patients: a longitudinal study. J Clin Psychiatry. 1990;51 Suppl:77-81.

[3] Zung W. Self-Rating Depression Scale. Arch Gen Psychiatry. 1965;12:63-70.

**Supplementary Figures/Tables**

**TABLE S1** Basic characteristics of 10 samples

| Group | ID | Sex | Age | Education | Family history | Smoker | Drinker | BMI(kg/m^2^) |
| --- | --- | --- | --- | --- | --- | --- | --- | --- |
| CHD group | A1 | Female | 57 | Primary | No | No | No | 29.30 |
|  | A2 | Female | 70 | Primary | No | No | No | 30.43 |
|  | A3 | Male | 61 | Primary | Yes | Yes | Yes | 20.70 |
|  | A4 | Male | 69 | Primary | No | No | No | 26.29 |
|  | A5 | Female | 78 | Junior | Yes | No | No | 23.44 |
| Control group | B1 | Male | 52 | Primary | No | Yes | No | 27.51 |
|  | B2 | Male | 56 | Junior | No | Yes | No | 28.73 |
|  | B3 | Male | 68 | Primary | No | No | No | 24.57 |
|  | B4 | Female | 63 | Primary | No | No | No | 20.08 |
|  | B5 | Female | 58 | Primary | No | No | No | 22.77 |
| *P* | 1.00* | 1.00* | 0.14 | 1.00* | 0.44* | 1.00* | 1.00* | 0.60 |

***** Fisher test

**TABLE S2** Primers used in the present study

| Name | Primer | Sequence (5ʹ–3ʹ) |
| --- | --- | --- |
| β-actin | forward | AGCGAGCATCCCCCAAAGTT |
|  | reverse | GGGCACGAAGGCTCATCATT |
| LncRNA ENST00000602339.1 | forward | GCTGAGGTCAGAATGGGAAG |
|  | reverse | CAACAGCTGCTATGTGCTTGA |
| LncRNA ENST00000565648.1 | forward | CATGTTGCGAACAAGTGTCA |
|  | reverse | CACTGTGCTGGCCTCAAATA |
| LncRNA ENST0000450016.1 | forward | GTGAGTCATGATCGCACCAC |
|  | reverse | TTTTACAACAGCTTCCGGGG |
| LncRNA ENST00000609755.1 | forward | GTGAAGTGGGCATCAAGGTAA |
|  | reverse | GAAGCGCAGTGGTGCTATCT |
| LncRNA ENST00000529247.1 | forward | AGGCTTCTTGGCCTTCTTCT |
|  | reverse | GGATGACGAGGATGATGACA |

**TABLE S3** siRNA sequence listing

| **Name** | **Primer** | **Sequence（5’-3’）** |
| --- | --- | --- |
| siRNA-1 | forward | GGGCACUCAUCCUAUAGUATT |
|  | reverse | UACUAUAGGAUGAGUGCCCTT |
| siRNA-2 | forward | CCAUAGAUGCCGGAUCAAATT |
|  | reverse | UUUGAUCCGGCAUCUAUGGTT |
| siRNA-3 | forward | GGUGAGAGCAAGACUUCAUTT |
|  | reverse | AUGAAGUCUUGCUCUCACCTT |
| NC | forward | UUCUCCGAACGUGUCACGUTT |
|  | reverse | ACGUGACACGUUCGGAGAATT |

**TABLE S4** Multiple logistic regression analysis of environmental factors and CHD risk

| Factors | Univariate analysis  OR (OR 95%CI) | Multivariate analysis^a^  OR (OR 95%CI) |
| --- | --- | --- |
| Family history of CHD | **1.596(1.123–2.267)** | **1.732(1.174–2.557)** |
| High-salt diet | **1.666(1.285–2.161)** | **1.543(1.153–2.066)** |
| Labour intensity  Moderate-intensity | 1.000 | 1.000 |
| Low-intensity | **1.610(1.232–2.104)** | **1.573(1.175–2.104)** |
| High-intensity | 0.783(0.577–1.064) | 0.716(0.508–1.010) |
| Physical activity  < 1 time/week | 1.000 | - |
| 1–2 times/week | 0.941(0.685–1.292) | - |
| 3–4 times/week | 0.684(0.417–1.123) | - |
| ≥5 times/week | 1.130(0.865–1.476) | - |
| Tobacco Smoking | **2.258(1.780–2.864)** | **2.487(1.883–3.285)** |
| Alcohol drinking | 1.084(0.786–1.496) | - |
| Tea drinking | 1.198(0.941–1.525) | - |
| Character  B type | 1.000 | - |
| A type | 1.180(0.905–1.537) | - |
| C type | 1.348(0.884–2.055) | - |
| D type | 1.677(0.717–3.923) | - |
| Depression (Normal) | 1.000 | 1.000 |
| Mild | **1.707(1.243–2.345)** | 1.305(0.899–1.895) |
| Moderate/Severe | 1.246(0.817–1.901) | 0.830(0.480–1.434) |
| Anxiety (Normal) | 1.000 | 1.000 |
| Mild | **1.970(1.309–2.967)** | 1.602(0.968–2.652) |
| Moderate/Severe | **2.571(1.115–5.925)** | **2.802(1.013–7.749)** |
| BMI  18.50–23.99 | 1.000 | 1.000 |
| <18.5 | 0.676(0.384–1.191) | 0.599(0.323–1.109) |
| ≥24.00 | **1.440(1.136–1.827)** | **1.511(1.166–1.985)** |

Abbreviations: CHD, coronary heart disease; OR, odds ratio; CI, confidence interval

^a^Adjusted for age, gender, marital status, and education level. Statistically significant variables in univariate analysis were selected for further multivariate analysis.

**TABLE S5** Basic characteristics of selected lncRNAs in microarray

| LncRNA ID | Fold change | *p* value | Regulation | LNCipedia name |
| --- | --- | --- | --- | --- |
| LncRNA ENST00000602339.1 | 4.252 | 0.007 | Up | MIR99AHG |
| LncRNA ENST00000565648.1 | 4.125 | <0.001 | Down | lnc-USP7-1 |
| LncRNA ENST00000450016.1 | 3.708 | 0.012 | Down | LINC01952 |
| LncRNA ENST00000609755.1 | 2.979 | 0.038 | Up | lnc-MICALL2-2 |
| LncRNA ENST00000529247.1 | 2.647 | 0.005 | Down | lnc-TIGD5-3 |

**
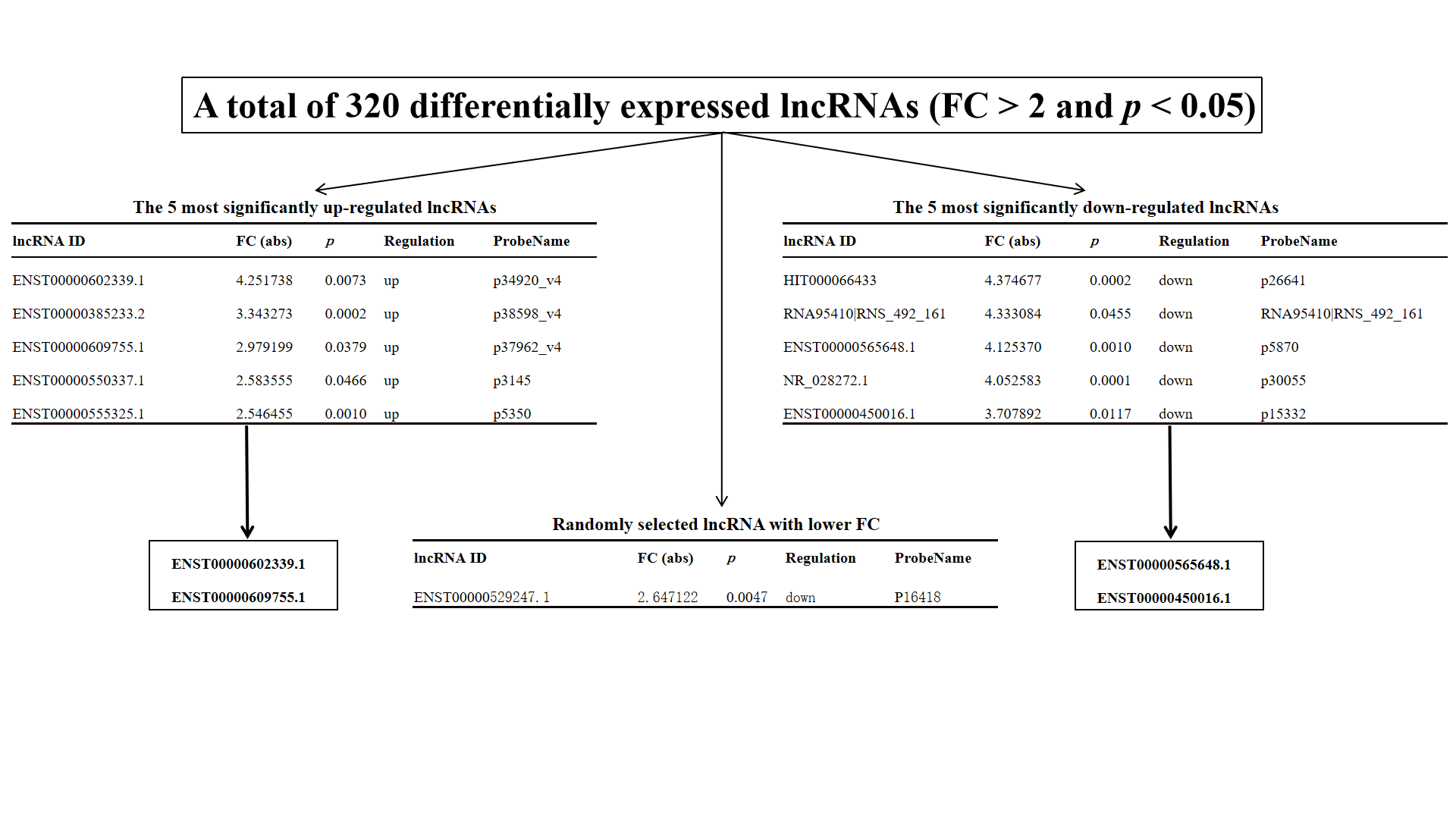
**

**FIGURE S1** Candidate lncRNAs screening process
